# A unifying biology of sex steroid-induced apoptosis in prostate and breast cancers

**DOI:** 10.1530/ERC-17-0416

**Published:** 2017-11-21

**Authors:** Philipp Y Maximov, Balkees Abderrahman, Ramona F Curpan, Yousef M Hawsawi, Ping Fan, V Craig Jordan

**Affiliations:** 1 Department of Breast Medical Oncology MD Anderson Cancer Centre, Houston, Texas, USA; 2 Institute of Chemistry Romanian Academy, Timisoara, Romania; 3 Department of Genetics King Faisal Specialist Hospital & Research Centre, Riyadh, Saudi Arabia

**Keywords:** estrogens, androgens, breast, prostate, apoptosis

## Abstract

Prostate and breast cancer are the two cancers with the highest incidence in men and women, respectively. Here, we focus on the known biology of acquired resistance to antihormone therapy of prostate and breast cancer and compare laboratory and clinical similarities in the evolution of the disease. Laboratory studies and clinical observations in prostate and breast cancer demonstrate that cell selection pathways occur during acquired resistance to antihormonal therapy. Following sex steroid deprivation, both prostate and breast cancer models show an initial increased acquired sensitivity to the growth potential of sex steroids. Subsequently, prostate and breast cancer cells either become dependent upon the antihormone treatment or grow spontaneously in the absence of hormones. Paradoxically, the physiologic sex steroids now kill a proportion of selected, but vulnerable, resistant tumor cells. The sex steroid receptor complex triggers apoptosis. We draw parallels between acquired resistance in prostate and breast cancer to sex steroid deprivation. Clinical observations and patient trials confirm the veracity of the laboratory studies. We consider therapeutic strategies to increase response rates in clinical trials of metastatic disease that can subsequently be applied as a preemptive salvage adjuvant therapy. The goal of future advances is to enhance response rates and deploy a safe strategy earlier in the treatment plan to save lives. The introduction of a simple evidence-based enhanced adjuvant therapy as a global healthcare strategy has the potential to control recurrence, reduce hospitalization, reduce healthcare costs and maintain a healthier population that contributes to society.

## Introduction

Despite advances in understanding the molecular biology of prostate and breast cancers, they are still the most frequently diagnosed cancers in men and women, in the United States. There is no completely effective preventative for either prostate or breast cancer. Advances in the chemoprevention of prostate cancer remain controversial (Bosland 2016) and none are approved by the Food and Drug Administration (FDA). As a result, there were 220,800 new cases of prostate cancer reported with 27,540 deaths (Siegel *et al*. 2015) in men. Advances in chemoprevention have been made in breast cancer (Jordan 2014*b*, 2016, 2017*a*, Cuzick 2015, Cuzick *et al*. 2016), but the task of implementation is not trivial (Kaplan *et al*. 2005, Owens *et al*. 2011, Smith *et al*. 2016). There were 231,840 new breast cancer cases reported in 2015, accounting for almost 29% of the total estimated female cancers (Siegel *et al*. 2015). Approximately, 40,290 deaths from breast cancer occurred in 2015 accounting for 14% of total deaths from cancers in women (Siegel *et al*. 2015). These figures present a major challenge in clinical research and for healthcare systems worldwide. Indeed, it is estimated that the incidence of breast cancer will increase by 50% from the level in 2011 for the combination of Indolent Lesion of Epithelial Origin (IDLE) and invasive disease by 2050 (Anderson *et al*. 2011). The increased survival of an aging population is the cause of the relentless rise in cancer. The goal of a cure remains. However, in practical terms, new affordable strategies are required for individuals affected by prostate or breast cancers to remain productive members of their families and society.

**Figure 1:**
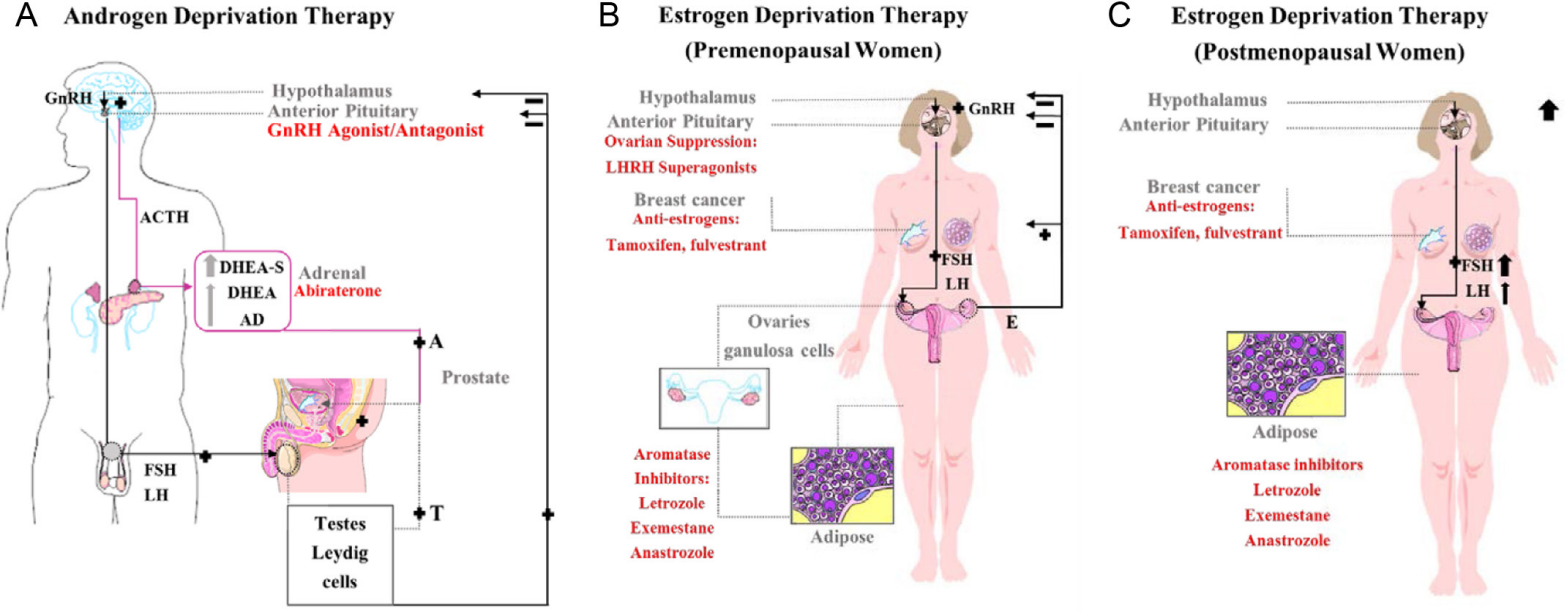
A schematic representation of the androgen and estrogen deprivation therapy in prostate cancer and pre- and postmenopausal women with breast cancer. (A) The hypothalamic–pituitary–gonadal and adrenal axis in prostate cancer with their therapeutic targets. The hypothalamus produces gonadotropin-releasing hormone (GnRH), which stimulates the adenohypophysis of the pituitary to produce adrenocorticotropic hormone (ACTH). This in turn, stimulates the adrenal gland cortex to produce androgens: dehydroepiandrosterone sulfate (DHEA-S) predominately, DHEA and androstenedione (AD) into the circulation. These androgens (A), alongside testosterone (T) from the testes, are converted in the prostate to their potent form, dihydrotestosterone (DHT). Dihydrotestosterone stimulates the growth of prostate cancer cells and exerts a negative feedback loop onwards to the hypothalamus and pituitary. Both, GnRH agonists/antagonists suppress LH production and cause a subsequent decline in serum testosterone to castrate levels. However, GnRH agonists (with chronic use) lead to the downregulation of GnRH receptors, whereas, GnRH antagonists usually cause an immediate blockade to the receptor. At the adrenal level, abiraterone inhibits adrenal androgen *de novo* steroidogenesis. At the prostate level, androgen receptor (AR) inhibitors are used and they have different mechanisms of action. For example, enzalutamide competitively inhibits the AR binding to DHT, inhibits nuclear translocation, and DNA and cofactor binding. Whereas, Bicalutamide is a highly selective, competitive and silent antagonist to the AR, which was also found to accelerate AR degradation. (B) The hypothalamic–pituitary–gonadal axis in premenopausal women with breast cancer and their therapeutic targets. The hypothalamus produces gonadotropin-releasing hormone (GnRH), which stimulates the adenohypophysis of the pituitary to produce luteinizing hormone (LH) and follicle-stimulating hormone (FSH). This in turn, stimulates the granulosa cells in the ovarian follicles to produce estrogen. However, FSH in particular stimulates the granulosa cells to produce inhibin, which suppresses FSH in a feedback loop and activin, a peripherally produced hormone that stimulates GnRH cells. Estrogen stimulates the growth of breast cancer cells, and exerts a negative feedback loop onwards to the hypothalamus and pituitary. Ovarian suppression can be achieved with LHRH superagonists such as goserelin, which is an analogue of LHRH, and a GnRH or LHRH agonist. Goserelin initiates a flare of LH production and ultimately leads to receptor downregulation. Antiestrogens can be estrogen receptor (ER) competitive blockers such as the Selective ER Modulators (SERMs, i.e. tamoxifen), or pure antiestrogens or what is known as a Selective ER Downregulators (SERDs, i.e. fulvestrant). Third-generation aromatase inhibitors (i.e. anastrozole, letrozole, exemestane) selectively block the aromatase enzyme system at the breast cancer level and therefore suppress estrogen synthesis. (C) The hypothalamic–pituitary–gonadal axis in postmenopausal women with breast cancer and their therapeutic targets. The differences from premenopausal women is that the ovarian follicles are depleted, therefore there is no active production of estrogen and progesterone. This leads to a dramatic increase in GnRH, an increase in FSH serum level relatively to that of LH through the feedback loops. Ovarian suppression is not used as a treatment option.

The sex steroid hormones i.e. androgens in men and estrogens in women play critical roles in the development and progression of prostate and breast cancers. Prostate cancer development relies on the androgen receptor (AR), whereas breast cancer development primarily relies on the estrogen receptor (ER). The majority of prostate and breast cancers are hormone dependent (Fig. 1). Antihormone therapies have had a profound impact in reducing the burden from breast cancer, worldwide (Jordan 2003, Santen *et al*. 2009*a*, Sledge *et al*. 2014). Here, we will address whether the lessons learned in breast cancer can be applied to prostate cancer therapy. Whether treatment strategies are the same or not for both diseases, resistance to antihormone treatments occurs in both prostate and breast cancers.

Currently, resistance to antihormone therapies in prostate and breast cancers are categorized as acquired resistance and *de novo* (intrinsic) resistance. It is considered that *de novo* resistance has the same mechanisms as the acquired resistance (Hoimes & Kelly 2010, Miller 2013), for the exception that these mechanisms are in place before the antihormone therapy is applied. We will focus on acquired resistance. In this review, we summarize the development of treatment approaches, the antihormonal agents used for the control of both diseases and the current understanding of the evolution of resistance to antihormonal therapies. We bring together these two major sex steroid-related diseases to define similarities and differences and compare and contrast treatments based on acquired antihormone resistance. We discuss the similarities of the phenomenon of sex steroid-induced apoptosis in both types of cancers after acquisition of antihormone resistance and explore the possibility that this new knowledge will have clinical applications. An innovative treatment approach that delivers affordable healthcare will save lives globally.

## Hormonal therapies for prostate and breast cancer

A diagnosis of advanced prostate cancer or breast cancer was a death sentence before 1940s, with patients dying within 1–2 years after diagnosis. Today, these same patients will have an earlier diagnosis, better care, but will still die within 3 years of diagnosis of stage IV disease. The number of patients with advanced prostate cancer has declined in the past 70 years, as early detection and diagnosis with proper treatment and monitoring has increased the 5-year survival rate up to 80–90% (Kirby *et al*. 2011). The change in the approach to treatment started when Professor Charles Huggins reported the response of metastatic prostate cancer (MPC) to androgen deprivation therapy (ADT), using surgical castration or high-dose synthetic estrogen therapy (Huggins & Hodges 1941). Diethylstilbestrol (DES) became a standard of care. Huggins won the Noble Prize in 1966 for developing a logical treatment strategy for prostate cancer with the ADT. Since then, ADT has been used as the gold standard for the treatment of MPC.

Earlier, but parallel, advances were reported for the treatment of advanced breast cancer in women. The initial experiment of oophorectomy (Beatson 1896) was proven to be effective in 30% of premenopausal breast cancer patients with metastatic breast cancer (MBC) (Boyd 1900). This was followed by a number of surgical ablation strategies and additive hormonal therapies for MBC (Kennedy 1965).

In the mid-1940s, Alexander Haddow (Haddow *et al*. 1944) was the first to discover that high doses of synthetic estrogens, including DES, could be used to treat postmenopausal women with MBC with a 30% response rate. Haddow’s (Haddow *et al*. 1944) clinical trial showed that only breast and prostate cancers were responsive, whereas all other types were not. Nevertheless, at that time, the mechanism of action was not understood (Haddow 1970). However, one important clinical fact did emerge. High-dose estrogen was only effective as an antitumor agent in MBC if used 5 years or more after menopause. High-dose estrogen therapy became the gold standard for the treatment of women with MBC until the introduction of tamoxifen 30 years later (Jordan 2003). The biologic mechanisms and therapeutic significance of estrogen therapy was, at that time, obscure. However, the development of models to discover mechanisms of what became the new biology of estrogen-induced apoptosis (Jordan 2008, 2015*a*) is now the central theme of this position paper.

The discovery of the AR in the late 1960s by three independent groups of Liao (Anderson & Liao 1968), Bruchovsky (Bruchovsky & Wilson 1968) and Mainwaring (1969), was an important breakthrough, as it triggered the search for androgen antagonists. Similar advances were made with the discovery of the ER in the early 1960s. Jensen first described the binding of radiolabeled estradiol in rat estrogen target tissues (Jensen & Jacobson 1962), and three years, later in 1966 Toft and Gorski identified the actual ER protein (Toft & Gorski 1966). Nevertheless, the therapeutic breakthrough of non-steroidal antiestrogens was focused on the modulation of fertility in rodents and women during the 1960s before the discovery of the ER (Jordan 1984, Lerner & Jordan 1990).

In the early 1970s, the first non-steroidal antiandrogen flutamide was discovered (Neri *et al*. 1972) and was approved in 1989 by the FDA for the treatment of prostate cancer. This discovery was followed by other non-steroidal antiandrogens including nilutamide (Raynaud *et al*. 1979) and bicalutamide (Furr *et al*. 1987), which were compared to castration in MPC patients in randomized trials. Results showed that antiandrogen drugs were better tolerated than castration (Chodak *et al*. 1995, Seidenfeld *et al*. 2000). However, they are inferior therapies in regard to overall survival (OS) and progression-free survival (PFS) (Chodak *et al*. 1995, Seidenfeld *et al*. 2000).

In 1971, an advance in physiology was made when Schally discovered the structure of the hypothalamic hormone known as the luteinizing hormone (LH)-releasing hormone (LHRH; called the gonadotropin-releasing hormone GnRH) (Schally *et al*. 1971). This led to an understanding of the sex steroid feedback control mechanisms orchestrated by the hypothalamo-pituitary axis (Fig. 1). Advanced prostate cancer patients who were treated with daily doses of the LHRH agonists had a 75% decrease in serum testosterone levels, a decrease or normalization of plasma acid phosphatase levels, and a significant decrease in cancer-associated bone pain (Tolis *et al*. 1982). In 1977, Schally received the Nobel Prize in Physiology and Medicine for discovering peptide hormone production in the brain. Many synthetic LHRH superagonists were subsequently developed for clinical use (Schally *et al*. 2000), such as buserelin, goserelin, leuprolide and nafarelin. Additionally, many LHRH antagonists have been developed and tested for the treatment of men with advanced prostate cancer such as orgalutran, cetrorelix and abarelix (Schally *et al*. 2000). An antagonist was considered to be necessary as the superagonists first stimulate gonadotropin release (which causes an androgen burst) before a desensitized and refractory state occurs. Estrogen has been used to treat prostate cancer by lowering gonadotropin levels and as a result androgen levels. Estrogen was evaluated successfully to block the stimulatory rise in gonadotropin caused by LHRH superagonists (Ahmann *et al*. 1987).

**Figure 2:**
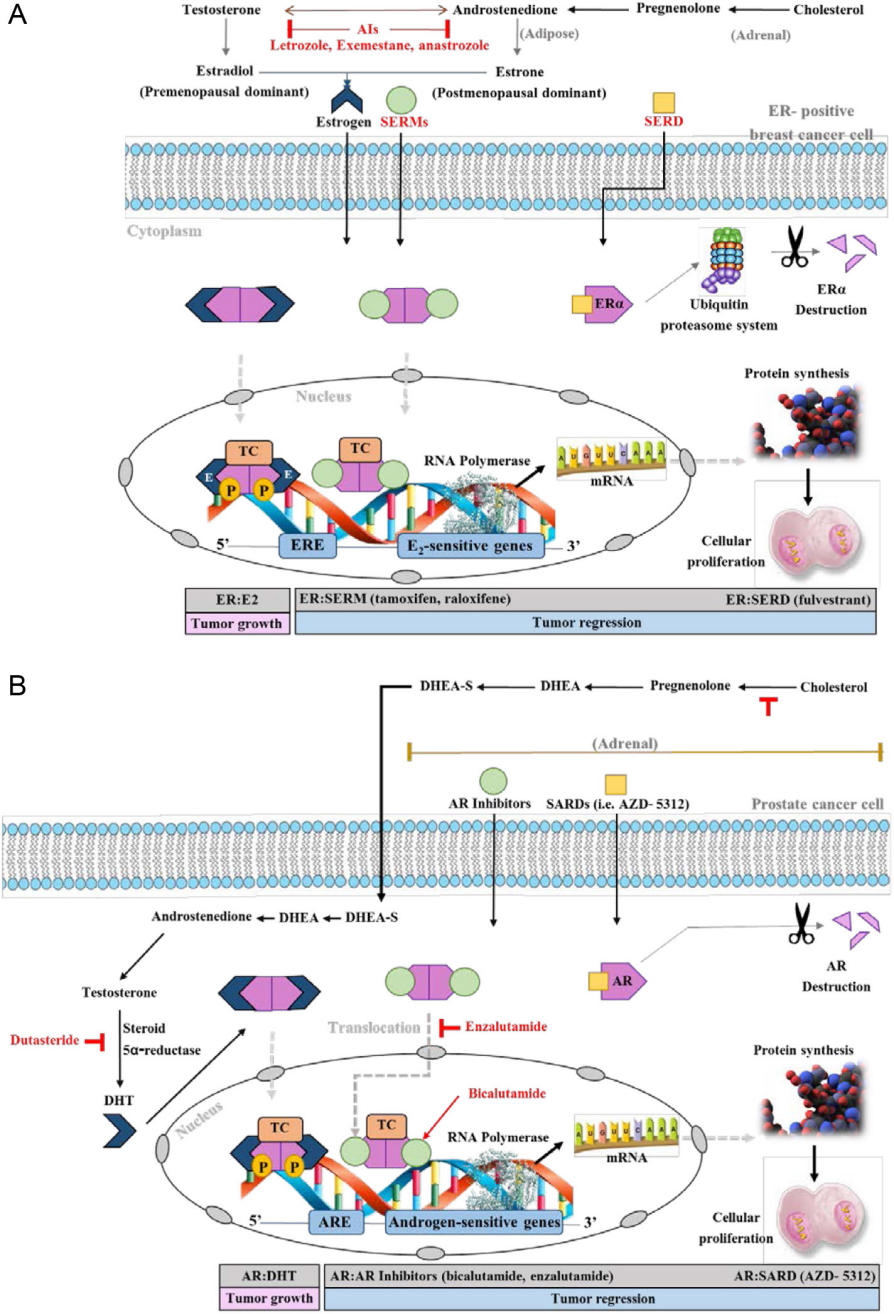
A schematic representation of the signal transduction pathways in ER-positive breast cancer cells and prostate cancer cells. (A) At the adrenal level, adrenal androgen *de novo* steroidogenesis occurs. Cholesterol is produced and converted to Pregnenolone with the aid of CYP11A1 enzyme. Pregnenolone is converted to dehydroepiandrosterone (DHEA) with the aid of CYP17A1. Finally, DHEA is converted to androstenedione (AD) with the aid of 3-β hydroxysteroid dehydrogenase enzyme. Then, AD is converted to testosterone via 17-β hydroxysteroid dehydrogenase. At the adipose tissue level, Both androstenedione and testosterone are converted with the aid of the aromatase enzyme system to estrone (predominant in postmenopausal women), and estradiol (predominant in premenopausal women), sequentially. Estrogen normally binds to the ER in the cytoplasm, the estrogen:ER complex translocates to the nucleus, gets phosphorylated, and binds to estrogen responsive elements (EREs) with the recruitment of coactivators. This creates a transcription complex (TC). This in turn, will initiate a cascade of protein synthesis and subsequent tumor proliferation through the activation of estrogen-sensitive genes. Whereas, SERMs:ER follows a similar pattern but recruits corepressors and inhibits protein synthesis; causing tumor regression. For SERDs, they bind to the ER causing an alien conformation. This leads to the destruction of the ER through the ubiquitin proteasome system; subsequently tumor regression. (B) At the adrenal level, adrenal androgen *de novo* steroidogenesis occurs. Cholesterol is produced and converted to Pregnenolone with the aid of CYP11A1 enzyme. Pregnenolone is converted to dehydroepiandrosterone with the aid of CYP17A1. Finally, DHEA is converted to DHEA-S with the aid of following enzymes: steryl-sulfatase (STS) and bile salt sulfotransferase. At the prostae level, DHEA-S in Leydig cells is converted back to DHEA via STS and then DHEA is converted to AD via enzyme 3β-HSD. Then, AD is converted to testosterone via enzyme AKR1C3, and finally to DHT via steroid 5α-reductase. Dihydrotestosterone normally binds to the AR in the cytoplasm, the DHT:ER complex translocates to the nucleus, gets phosphorylated, binds to androgen responsive elements (AREs) with the recruitment of coactivators. This creates a transcription complex (TC). This in turn, will initiate a cascade of protein synthesis and subsequent tumor proliferation through the activation of androgen-sensitive genes. Whereas, AR inhibitors:AR complex follows a similar pattern but recruits corepressors and inhibits protein synthesis; causing tumor regression. For SARDs, they bind to the AR causing the degradation of the receptor; subsequently tumor regression. Androgen receptor inhibitors vary in their mechanisms of action. For example, enzalutamide competitively inhibits the AR binding to DHT, inhibits nuclear translocation of AR, and DNA and cofactor binding. Whereas, bicalutamide is a highly selective, competitive and silent antagonist to the AR, which was also found to accelerate AR degradation. Abiraterone inhibits CYP17A1 and subsequently adrenal androgen *de novo* steroidogenesis. Dutasteride is a 5α-reductase inhibitor that blocks testosterone conversion into DHT.

Fernand Labrie (Labrie *et al*. 1986) was one of the pioneers who developed the idea of a complete androgen blockade using combination antiandrogen therapy with flutamide and LHRH agonists or surgical castration in patients with MPC increasing the PFS and OS. Crawford and colleagues (Crawford *et al*. 1989) demonstrated that the combination of flutamide and leuprolide resulted in a slightly longer PFS. As a result, many physicians in the United States shifted toward combined androgen blockade as initial therapy for advanced prostate cancer. The signaling pathways of the AR and mechanism of action of different antiandrogens is depicted in Fig. 2.

In contrast, breast cancer treatment strategies followed a separate path with an early move from the treatment of MBC to adjuvant therapy following breast surgery. A key factor in the differences in the treatment strategies of prostate and breast cancer is the fact that the majority of breast cancer occurs after menopause when there is no hypothalamo–pituitary–ovarian communication to alter estrogen levels (Fig. 1). By contrast, a recognized menopause does not occur in men and, as a result, hormonal communication from the pituitary to the testicular target remains. Currently clinical strategies are being defined and refined to address breast cancer treatment in the premenopausal patients (Abderrahman & Jordan 2016, Rossi & Pagani 2017).

The ER became the target for tamoxifen to treat breast cancer (Jordan & Koerner 1975) based on the National Cancer Institute consensus conference in Bethesda in 1974 on ERs in human breast cancer (McGuire *et al*. 1975). Treatment strategies in the 1970s for breast cancer proposed, long-term adjuvant antihormone therapy (Jordan 1978, 2014*b*) and the possibility of chemoprevention (Jordan 1976). These treatment strategies were proposed before tamoxifen was approved for the treatment of MBC in the United States (December 29th, 1977).

The actual development of tamoxifen was not initially a major priority by the pharmaceutical industry, but dependent upon chance and the investment in young scientists (Jordan 2006, 2015*b*). Tamoxifen’s withdrawal from clinical development and resurrection in the 1970s with a clear strategic plan for the development of the medicine was the key to success (Jordan 2006, 2014*b*). Tamoxifen became the standard for antihormonal therapy of ER+ MBC (Furr & Jordan 1984, Jordan 2003, 2006). Five years of adjuvant treatment with tamoxifen improved clinical outcome compared to shorter adjuvant therapy (EBCTCG 1998), and for more than a decade, 5 years of adjuvant tamoxifen (Davies *et al*. 2011) (or aromatase inhibitors, AIs) was the standard of care for ER+ breast cancer. Tamoxifen was the first medicine, in a new group of medicines called the selective estrogen receptor modulators (SERMs) (Maximov *et al*. 2013). Ultimately, tamoxifen was the first antiestrogen to be approved by the FDA for the prevention of breast cancer in women (Jordan 2003).

Another approach to treat breast cancer inhibits the aromatase enzyme system (CYP19) that catalyzes estrogen biosynthesis in postmenopausal women. This group of medicines is called the AIs, and these are currently used for the treatment of postmenopausal breast cancer patients. Aminoglutethimide was the first Al introduced, which has an efficacy in MBC patients (Lipton & Santen 1974). Nevertheless, all the AIs used in the early 1970s were not specific for CYP19 and showed side effects with depression of adrenal function. Glucocorticoids needed to be used to compensate (Santen *et al*. 1981). As a result, the first-generation AIs (aminoglutethimide and testololactone) were not suitable for adjuvant treatment (Cocconi 1994). The breakthrough occurred in the late 1970s with the discovery of the first specific inhibitor of the aromatase system, 4-hydroxy androstenedione (Brodie *et al*. 1977, 1979). This compound, known as formestane, demonstrated clinical efficacy in MBC (Coombes *et al*. 1984, Goss *et al*. 1986, Dowsett *et al*. 1987). Again, regrettably, the medicine was unsuitable for adjuvant trials of ER+ breast cancer, because it was an injectable. Soon after the development of the third generation of Als, (anastrozole, letrozole and exemestane) with lower toxicity, the Als became the adjuvant endocrine treatment of choice for the ER+ postmenopausal breast cancer patients (Dowsett *et al*. 2010). The signaling pathways of the ER and mechanism of action of different antiandrogens are depicted in Fig. 2.

## Current treatment strategies for prostate and breast cancers

Various parameters, such as the tumor volume and the pathological grade, have been correlated with prostate cancer malignancy (Bostwick *et al*. 2000). A strong correlation with an excellent prognosis was evident in prostate cancer presenting with a high percentage of AR-positive cells (Barboro *et al*. 2014). Prostate cancer that is AR negative is very rare; therefore, little attention has been given to this subtype. The aggressiveness of prostate cancer is based on the Gleason score, a system based on pathological grade. In prostate cancer, the lowest Gleason score sum found in a tumor biopsy is 6, which are low grade or well differentiated, less aggressive with slow growth and limited invasion and metastasis. The Gleason score sum of 8–10 are found in high-grade tumors, poorly differentiated, tending to be aggressive and quickly grow and spread. Gleason score sum of 7 is called intermediate grade and is found in moderately differentiated tumors.

**Figure 3:**
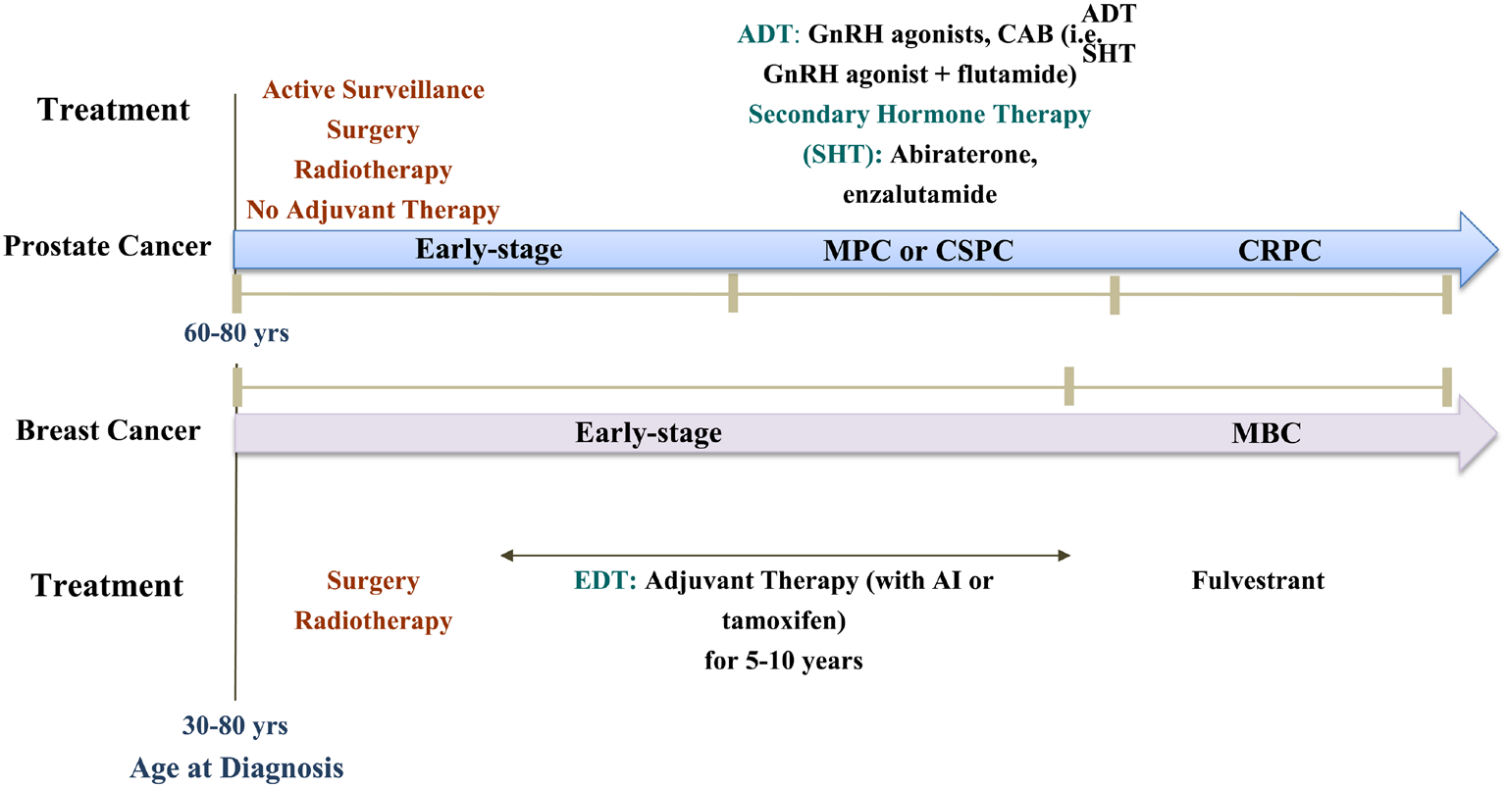
A schematic representation of the treatment paradigms used clinically for breast and prostate cancers. (A) Early-stage prostate cancer (PC) is usually approached with active surveillance, local treatments such as: surgery and radiation therapy. Hormone therapy can be given for early-stage PC men if they were at high-risk, or if they cannot undergo surgery or radiation therapy. The newer treatments for early-stage PC are: Intensity-Modulated Radiation Therapy, Proton beam therapy, and Cryosurgery. If early-stage PC progresses to metastatic PC (MPC) or what is known as castration-sensitive PC (CSPC), it will be treated with androgen deprivation therapy (ADT) using GnRH agonists, or complete androgen blockade (CAB) using a GnRH agonist plus flutamide for example, or secondary hormone therapy (SHT) using abiraterone, or enzalutamide as examples. If CSPC progresses to castration-resistant PC (CRPC), it will be treated with ADT or SHT. About 60% of PC is diagnosed in men >65, with 97% in men age >=50. The median age at the time of diagnosis in the U.S. is about 66. (B) Early-stage BC can be treated with local treatments such as: surgery and radiotherapy or systemic treatments such as: hormone therapy. What sets early-stage BC treatment apart from prostate cancer is adjuvant therapy with tamoxifen or AIs for 5–10 years. If early-stage BC progresses to metastatic BC (MBC), one therapeutic option is fulvestrant. Breast cancer rates increase after age 40 and are highest in women >70. The median age of diagnosis of BC for women in the U.S. is 62.

Since prostate cancer is an indolent disease, the majority of men diagnosed will not be treated with any type of therapy. It was found that the majority of men with prostate cancer have lower prostate cancer-specific mortality rates and are more likely to die from age-related comorbidities (Lu-Yao *et al*. 2009, Albertsen *et al*. 2011). However, if prostate cancer is graded as aggressive, then surgery and sometimes adjuvant radiotherapy are the therapies of choice. Radical prostatectomy alone in men with localized prostate cancer has a 7-year recurrence-free survival (RFS) of approximately 70% (Kattan *et al*. 1999) and the biochemical PFS of approximately 50% (Bolla *et al*. 2005). However, application of immediate adjuvant radiotherapy can further significantly increase clinical PFS (Bolla *et al*. 2005). If the disease has progressed in spite of primary therapies, has metastasized or is an advanced poor prognosis or/and high-grade tumor only then is hormonal therapy applied. Recurrent tumors that are nonmetastatic or for locally advanced tumors (tumors that have spread to nearby tissue or local lymph nodes) are sometimes treated with adjuvant hormonal therapy concomitantly with adjuvant radiotherapy. This can further increase PFS and OS, especially if applied at earlier time points (Fleshner *et al*. 2008, Payne & Mason 2011, Omrcen *et al*. 2015, Shipley *et al*. 2017). Current treatments strategies for prostate cancer are summarized in Fig. 3.

By contrast, breast cancer is a highly heterogeneous tumor with different malignant subtypes. Prat and Perou (2011) used gene expression profiling to classify breast cancer into subtypes based on the expression of the main receptors, ER, progesterone receptor (PR) the erythroblastosis oncogene (ErBB2, HER2/neu) and the AR: Luminal A (ER+, PR+, HER2− and low Ki-67, low grade), Luminal B (ER+, PR+, HER2+/−, high Ki-67 and high grade), human epidermal growth factor receptor 2 (ER−, PR− and HER2+), basal-like or triple-negative (TNBC) (ER−, PR− and HER2−), claudin-low (often TNBC with low expression of cell-to-cell contact proteins and E-cadherin, in particular, with infiltration of lymphocytes), Luminal ER−/AR+ (AR+ and respond to antihormonal therapy with antiandrogens (Gucalp & Traina 2016)) and normal-like (ER+, PR+, HER−, low Ki-67 and normal like) breast cancers. Patients with ER+ early-stage breast cancer account for about 75% of breast cancer cases (Harvey *et al*. 1999). Though the primary therapies for early-stage breast cancers, regardless of their subtype are surgery and radiotherapy, long-term hormonal adjuvant therapy is used in most cases with ER+ breast cancers. The first FDA-approved antiestrogen tamoxifen is usually prescribed to premenopausal patients as they have a very low risk of developing endometrial cancer as a side effect from long-term tamoxifen treatment. The Early Breast Cancer Trialists’ Collaborative Group in 2011 confirmed that five years of using tamoxifen as adjuvant treatment reduced the risk of death and reduced the 15-year recurrence risk by 40% (Davies *et al*. 2011). The benefits of the 10-year extended therapy with tamoxifen were presented in two studies in 2013 with the 15-year follow-up of the Adjuvant Tamoxifen: Longer Against Shorter (ATLAS) trial (Davies *et al*. 2013) and the Adjuvant Tamoxifen To Offer More (aTTom) (Gray *et al*. 2013) trial. The outcome of the ATLAS trial is that, ER-positive patients with extended tamoxifen therapy reduced the risk of breast cancer recurrence, mortality and reduced overall mortality (Davies *et al*. 2013). The outcome of the aTTom trial was similar to the ATLAS trial confirming that, continuing tamoxifen treatment in ER-positive breast cancer patients for 10 years rather than just 5 years leads to further reductions in recurrence and subsequent decrease in mortality. The documentation for the clinical characteristics of high-risk patients eligible for extended tamoxifen therapy (>5 years) have recently been published (Pan *et al*. 2016).

For postmenopausal women with thromboembolic or no osteoporotic comorbidities, AIs are usually prescribed. Anastrozole was the first of the third-generation AIs used in a clinical trial called the Tamoxifen Alone or Combination (ATAC) trial (Baum *et al*. 2002). Anastrozole has some advantages over tamoxifen as a first-line adjuvant treatment for early breast cancer in postmenopausal patients. Results for the combination treatment are the same as tamoxifen alone (Baum *et al*. 2002, Cuzick *et al*. 2010). This was to be expected. A rule of pharmacology is that a partial agonist (tamoxifen) that binds to a receptor when combined with a therapy that removes the ligand from the body produces a response of the partial agonist alone. In 2010, a meta-analysis (Dowsett *et al*. 2010) was performed and demonstrated the superiority of 2–3 years of tamoxifen followed by an AI for 2–3 years over 5 years of tamoxifen alone. Other clinical trials, referred to as the Breast International Group (BIG 1–98) (Breast International Group 2005) and the adjuvant tamoxifen and exemestane in early breast cancer (TEAM-trial) (van de Velde *et al*. 2011), were designed to address the question whether AIs would be superior to tamoxifen or not after 2–3 years of tamoxifen followed by switching to an AI for five years, showed no significant decrease in disease-free survival (DFS) or the RFS. A meta-analysis of individual data from postmenopausal patients with early-stage ER-positive breast cancers comparing 5 years of AIs against 5 years of tamoxifen or switching to an AI up to year 5 after 2–3 years of tamoxifen compared to 5 years of tamoxifen or an AI alone has shown that AIs have a significantly more favorable recurrence rates (RR) than tamoxifen by 30% (Early Breast Cancer Trialists’ Collaborative Group 2015). AIs also caused more bone fractures, but fewer cases of endometrial cancers than tamoxifen (Early Breast Cancer Trialists’ Collaborative Group 2015).

Currently, the period of adjuvant tamoxifen/AI treatment is extended up to 10 years based on the National Cancer Institute of Canada Clinical Trials Group (NCIC CTG MA17), which showed the superiority of 5 years of tamoxifen followed by five years of letrozole compared to 5 years of tamoxifen alone (Goss *et al*. 2003). Recently, Goss and colleagues found in the MA17 extension using an additional 5 years of AI for a 10 years total significantly increases the rates of DFS and decreases the incidence of contralateral breast cancer but the rate of OS was not increased (Goss *et al*. 2016). It should be noted, however, that in the ATLAS trial (Davies *et al*. 2013) and the combined analysis of ATLAS and aTTom trials mortality did not decrease significantly during extended adjuvant therapy but only a decade after extended therapy. As tamoxifen is a competitive inhibitor of estrogen action at the ER (Jordan 1984), and is not cytotoxic, it is suggested that decreases in mortality occur by cell selection and subsequent estrogen-induced apoptosis from the woman’s own estrogen (Jordan 2014*a*, 2015*a*). Breast cancer remains the only cancer with an option of long-term adjuvant antihormone therapy proven to save lives. Current treatment strategies for breast cancer are summarized in Fig. 3.

The application of antihormone therapy is crucial in ER+ breast cancer as it is able to reduce the recurrence of breast cancer at least by half, and, unlike prostate cancer, which is an indolent disease, breast cancer will progress faster and recur without treatment. All prostate cancer patients and half of breast cancers develop acquired resistance to antiestrogen therapy.

## Understanding acquired resistance to hormonal therapies in prostate cancer

ADT is the primary therapy for prostate cancers that are classified as an aggressive type (high Gleasson score sum), advanced or locally advanced. The average duration of clinical responses to antiandrogen therapies in advanced prostate cancer is 12–18 months after which practically all patients evolve to castration-resistant prostate cancer (CRPC) tumor phenotype. CRPC is characterized by consistent elevation of prostate-specific antigen (PSA) despite ADT and/or metastases. It is estimated that 10–20% of all non-advanced prostate cancer patients will progress to CRPC after surgery or radiotherapy (Kirby *et al*. 2011).

Currently multiple examples exist for the molecular mechanisms of antihormone resistance in prostate cancer with an analogous classification for breast cancer. Each mechanism or their combinations may have clinical applications in individual cases. The mechanisms of acquired resistance to antihormone therapies for prostate cancer can be categorized based either on the dependence on the AR or dependence on the ligand (Table 1).

**Table 1:** Mechanisms of resistance to antihomrone treatments are similar in both prostate and breast cancer cancers.

**Category**	**Mechanisms**
Ligand-dependent, receptor-dependent	hypersensitivity of the receptor to the ligand due to point mutations increased receptor expression increased transcriptional activity of the receptor due to changes in coregulators and corepressors levels increased levels of endogenous or circulating ligand
Ligand-independent, receptor-dependent	gain-of-function mutations in the receptor cross-talk mechanisms with other growth factor pathways
Bypass pathway (ligand-independent, receptor-independent)	deactivation of tumor suppressor pathways high expression of anti-apoptotic and low expression of pro-apoptotic molecules activation of cell proliferation survival pathways
Hormone receptor negative, ligand-independent	activation of growth factor receptor pathways employment of other types of hormone receptors

They have been categorized by their dependence on the hormone receptors or hormones themselves.

### Ligand-dependent and receptor-dependent mechanisms of resistance

**Figure 4:**
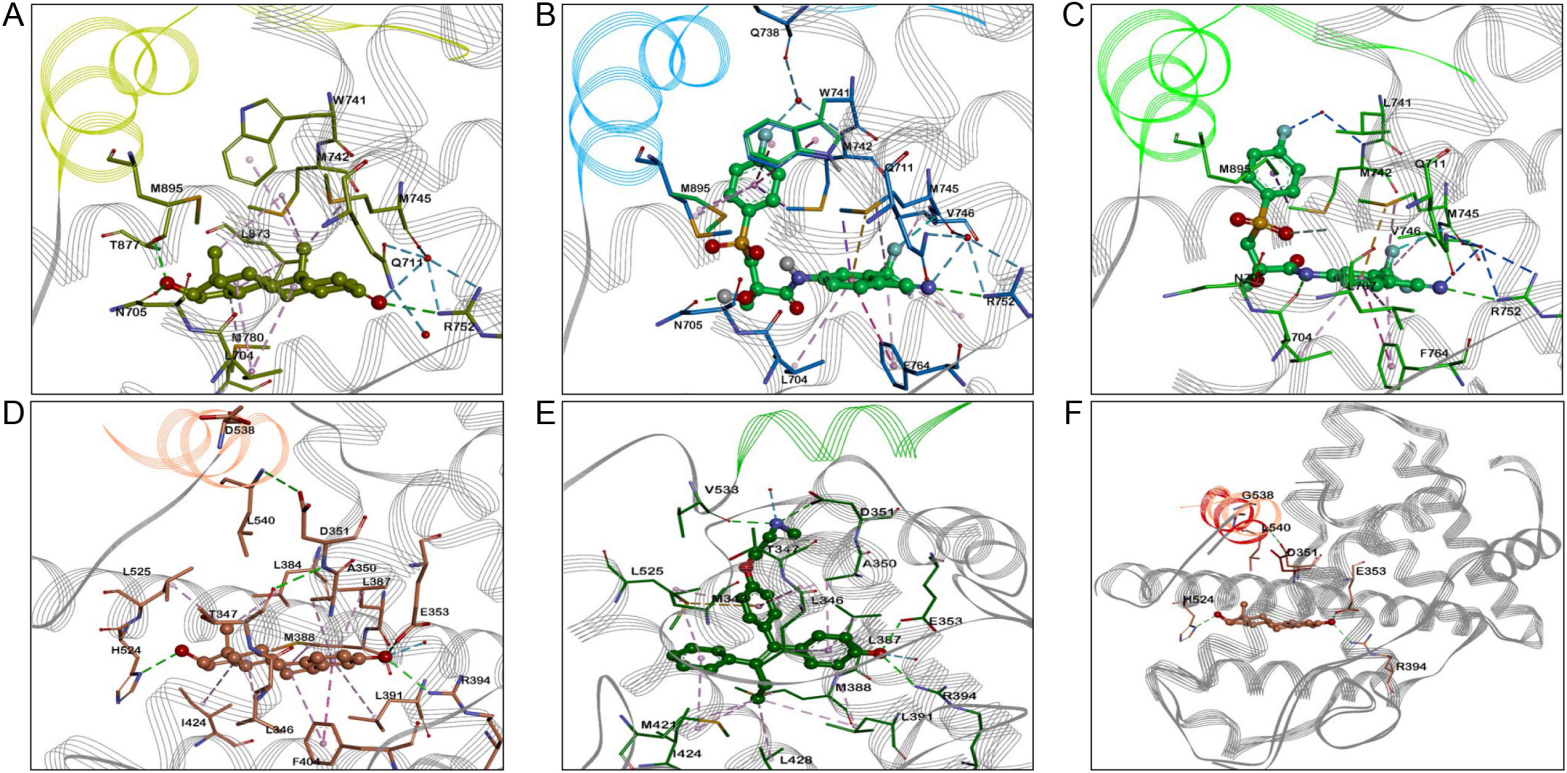
Molecular modeling of the wild-type and mutant ER and AR bound with agonists and antagonists. (A) wtAR:DHT LBD complex (PDB ID: 3L3X); (B) the best docking pose of the wtAR:bicatulamide complex (PDB ID: 3RLJ), obtained via flexible docking (the experimental structure used for docking was selected based on the 3D similarity between bicatulamide and the available ligands co-crystalized with AR WT, thus the experimental structure 3RLJ was selected due high similarity between the native ligand, S-22 and bicatulamide). The major interactions are shown in dashed lines and colored as follows: hydrophobic interactions in lavender, pi-pi interactions in purple, water-mediated H-bonds are shown in blue, and classical H-bonds are depicted in green.; (C) T741L AR mutant:bicatulamide LDB complex (PDB ID: 1Z95), helix 12 is colored in green and the major interactions are shown in dashed lines and colored as follow: hydrophobic interactions in lavender, pi-pi interactions in purple, water-mediated H-bonds are shown in blue, and classical H-bonds are depicted in green; (D) wtER:E2 LBD complex (PDB ID: 1GWR); (E) wtER:endoxifen LBD complex; (F) Superposition of E2 D538G mutant with ERα D358G apo LBD structures (helix 12 is shown in red for apo conformation and pink in the E2 bound mutant structure). The major interactions are shown in dashed lines and colored as follow: hydrophobic interactions in lavender, pi-pi interactions in purple, water-mediated H-bonds are shown in blue, and classical H-bonds are depicted in green.

Mutations in the AR are found in almost 30% of metastatic CRPC (mCRPC) (Navarro *et al*. 2002, Waltering *et al*. 2012). The majority of mutations in the AR are identified in the metastases, rather than in the primary tumors (Marcelli *et al*. 2000) and may enable the AR to bind some antiandrogens, such as flutamide and bicalutamide, that act as AR agonists and fuel tumor cell growth (Buchanan *et al*. 2001, Bohl *et al*. 2005*a*,*b*). We have performed molecular dynamics modeling to demonstrate the difference in the conformations of the ligand-binding domains (LBD) of the wild-type (wt) AR bound with an agonist (DHT) and antagonist bound with wtAR and a mutant AR found in CRPC (Bohl *et al*. 2005*a*) (Fig. 4). The modeling results show that, when compared with the wtAR:DHT complex (Fig. 4A), the helix 12 of the mutantAR:bicalutamide complex closes over the LBD of the receptor, which provides agonist conformation of the AR and its subsequent activation (Fig. 4C). It should be noted that the precise mechanism of antiandrogen action at the LBD of the AR remains unclear (Bisson *et al*. 2008, Duke *et al*. 2011, Tan *et al*. 2015). Activating mutations at the ER can explain the phenomenon of antiandrogen withdrawal syndrome, when the termination of therapy with antiandrogens is followed by regression of tumors (Hara *et al*. 2003).

Besides various mutations of the AR that contribute to the endocrine resistance in CRPC, a new role of membrane-associated AR isoforms in CRPC is emerging. Membrane-bound ARs have been identified in LNCaP cells and in hormone-insensitive DU145 cells and are associated with rapid non-genomic hormone responses in cells (Papakonstanti *et al*. 2003, Papadopoulou *et al*. 2008*a*,*b*). However, very little is known about the significance of the membrane-bound ARs in CRPC, but recent report identifies an AR splice variant called the AR8 that is shown to be associated with castration resistance in prostate cancer (Yang *et al*. 2011). Overexpression of the AR8 isoform increases the association of the receptor with the EGFR in CRPC cells and promotes cell proliferation and survival (Yang *et al*. 2011).

The AR in CRPC cells can become hypersensitive to low doses of androgens. This hypersensitivity is associated with mutations in the AR itself, leading to an increased sensitivity of the receptor to low concentrations of circulating androgens (Gregory *et al*. 2001*b*). Additionally, overexpression of the AR can be another AR-dependent mechanism that creates hypersensitivity. Indeed, it was shown that 30% of CRPC tumors overexpress the AR at high levels in the cells and 80% of patients show an elevated gene copy number (Feldman & Feldman 2001, Waltering *et al*. 2012), which may be a result of selection of cell populations with high levels of the AR under androgen deprivation pressure (Rau *et al*. 2005). An increase in the AR expression is associated with the amplification of the AR gene and in some cases attributed to polysomy of the X chromosome (Ropke *et al*. 2004). However, androgen action is not dependent upon the AR alone but is modulated by coregulators. The levels of expression of these coactivators, particularly SRC1 and SRC2, are higher in poorly differentiated prostate tumors or in recurrent prostate cancers and provide cells with higher AR activity in a low-dose androgen environment (Fujimoto *et al*. 2001, Gregory *et al*. 2001*a*). Additional AR-specific coactivators of note have been identified: ARA70 increases the AR activity and even facilitates the binding of estradiol to the AR (Yeh *et al*. 1998), FKBP51 stabilizes the AR with HSP90 heat-shock protein complex and facilitates the binding of androgens (Ni *et al*. 2010), and TRIM24 increases AR transcriptional activity (Groner *et al*. 2016).

Long-term antiandrogen therapy can affect the hypothalamo–pituitary axis as a negative feedback loop in men, leading to a compensatory increase of circulating androgens (Rau *et al*. 2005). This, by itself, can activate the AR by the law of mass action, but testosterone is not the physiologic ligand as it is required to be converted to dihydroxytestosterone (DHT) in the prostate cancer cell. Increased 5α-reductase enzyme activity contributes to the increase of endogenous androgens in the tumor. This results in the selection of CRPC cells able to convert androgen to endogenous DHT to produce a growth advantage (Navarro *et al*. 2002, Titus *et al*. 2005, Chang *et al*. 2014). A polymorphism in the 5α-reductase gene is noted in men of African-American descent, which is responsible for higher enzymatic activity in prostate cancer cells, as well as in prostate cancer cases with bad prognosis (Ruijter *et al*. 1999). In fact, the intratumoral levels of androgens can be as high as 40% above the baseline levels before ADT (Nishiyama *et al*. 2004). Recently another polymorphism in HSD3B1, which encodes 3β-hydroxysteroid-dehydrogenase-1 has been identified in a retrospective study of CRPC as a factor that correlates with an increased DHT synthesis (Hearn *et al*. 2016).

### Ligand-independent and receptor-dependent mechanisms of resistance

Most mutations are point gain-of-function mutations and are mostly located in the LBD of the AR and allow other sex steroids, such as glucocorticoids to bind to the AR and activate it (Zhao *et al*. 2000). Resistance to abiraterone was demonstrated in some mCRPC tumors with mutated AR (Cai *et al*. 2011, Chen *et al*. 2015). Additionally, alternative AR mRNA splice variants occur that generate constitutively active AR proteins (Dehm *et al*. 2008, Watson *et al*. 2010, Bubley & Balk 2017).

Increased expression of certain growth factors are associated with increased activity of the AR in mCRPC as well. The subversion of the AR transcriptional activity via growth factor receptor-mediated growth is called the cross-talk. Epidermal growth factor (EGF), keraticocyte growth factor (KGF) and insulin-like growth factor 1 (IGF-I) can activate the AR and can be reversed by antiandrogens (Culig *et al*. 1994). Tyrosine kinase receptors, such as HER2, which is highly expressed in CRPC cells, can also activate the AR via phosphorylation, through activation of the MAPK and the Akt pathways (Lin *et al*. 2001). The growth factor IL-6 is responsible for the progression of CRPC. This occurs by increasing AR activity by 50% more than observed with DHT alone (Culig *et al*. 2002). Resistance to growth inhibition occurs through the MAPK and STAT3 pathways, induces autophosphorylation of HER2 to activate the AR-mediated cascade independent of the ligand (Chen *et al*. 2000, Culig *et al*. 2002). High expression levels of the AR mRNA are maintained by NF-κB in CRPC cells, which sustains high AR protein levels (Zhang *et al*. 2009).

### Bypass pathway

One of the mechanisms of resistance involves recruitment of cellular survival pathways in CRPC, but the tumors still express the AR. However, the tumor cells do not require active AR to proliferate and survive. This mechanism is called the bypass pathway. Over expression of the antiapoptotic genes like Bcl-2, Bcl-xL and NF-kB (Gleave *et al*. 1999) are characteristic of the bypass pathway. Activation of other cell survival signaling pathways like PI3K/Akt has also been examined and linked with CRPC progression (Taylor *et al*. 2010). Mutations in tumor suppressor genes, like PTEN, also play a role in hormone resistance and allow the cells to rapidly progress though the cell cycles (Li *et al*. 1997, Mulholland *et al*. 2011, Edlind & Hsieh 2014). Mutated BRCA1 and BRCA2 tumor suppressor genes that are strongly associated with breast cancer incidence and progression have also been shown to be present in CRPC cells and associated with progression of prostate cancer cells to a CRPC phenotype (Rosen *et al*. 2001, Kote-Jarai *et al*. 2011, Leongamornlert *et al*. 2012, Robinson *et al*. 2015). Several other proteins have been identified that are associated with AR bypassing and progression and survival of therapy-resistant CRPC, such as TWIST1, DKK3 and VAV3 (Marques *et al*. 2010).

Recently, other possible contributing factors to the progression of CRPC disease were identified. Some estrogens are synthesized in males and the ERα is expressed in CRPC cells. The activation of the ERα stimulates proliferation and migration of the tumor cells (Attia & Ederveen 2012, Mishra *et al*. 2015). The estrogen-related receptor (ERR) induces bone metastases and activation of VEGF-A, WNT5A and TGFβ1 in mCRPC cells (Fradet *et al*. 2016). The glucocorticoid (GR) and the mineralocorticoid receptors (MR) have been associated with the progression of CRPC and being able to substitute and bypass the blocked AR (Arora *et al*. 2013). It appears that the GR and the AR have an array of common response genes due to homologous DNA-binding domains of the receptors (Sahu *et al*. 2013, Grindstad *et al*. 2015).

### Ligand-independent mechanisms of resistance with the loss of the AR

Antiandrogen resistance in prostate cancer can also be ligand independent and AR negative. Loss of expression of the AR in CRPC has been recorded in 30% of cases (Suzuki *et al*. 2003) due to hypermethylation of the AR gene (Jarrard *et al*. 1998). This epigenetic deregulation of the AR expression is much more common for the CRPC compared to only 10% of *de novo* hormone-resistant prostate cancers (Suzuki *et al*. 2003). Selected cells, with loss of the AR expression after antihormonal therapy, have adapted and ‘hijacked’ pathways enabling them to grow using other growth stimulatory pathways and even employ other hormone receptor pathways.

## Understanding acquired resistance to hormonal therapies in breast cancer

The mechanisms of antihormone resistance in breast cancer cells are very similar to the mechanisms in CRPC (Table 1) (Rau *et al*. 2005, Risbridger *et al*. 2010).

### Ligand-dependent and receptor-dependent mechanisms of resistance

The hypersensitivity of breast cancer cells to low doses of estrogens during estrogen ablation therapy has been associated with increased levels of ER expression. For instance, the ER protein levels were shown to be higher in long-term estrogen-deprived (LTED) MCF-7 cells by as high as 10-fold (Katzenellenbogen *et al*. 1987, Welshons & Jordan 1987). This can also happen in estrogen depletion with tamoxifen treatment (Berstein *et al*. 2004). One of the possible pathways of such hypersensitivity to estrogens was explained by a non-genomic activity of the ER, when it phosphorylates Shc, which in turn binds to signaling proteins Grb-2 and Son of Sevenless (SoS). As a result, this activates MAPK/ERK via Ras and Raf and promotes the phosphorylation of the ER at the AF-1 motif and activation of the receptor (Santen *et al*. 2003). The increased transcriptional activity of the ER can also be upregulated by overexpressed coactivators. Estrogen receptor coactivator SRC3 is the most important for breast cancer as its expression is restricted to only a few tissues, including the breast (Suen *et al*. 1998). Clinical studies (Osborne *et al*. 2003, Alkner *et al*. 2016) noted that high levels of SRC3 coactivator were associated with worse outcomes in tamoxifen-treated breast cancer patients. Low corepressor expression has been described in tamoxifen-resistant tumors and has been reviewed elsewhere (Legare & Basik 2016). Asides from the levels of the ER protein and its activity modulating cofactors, high levels of circulating and intratumoral hormones can also provide antihormone resistance. As tamoxifen binds and blocks the ER in breast tumor cells, it can also bind to the ER in pituitary gland and hypothalamus and disrupt the negative feedback loop. Tamoxifen induced an elevation of the circulating levels of estrogens secreted from the ovaries by increasing gonadotropin-releasing hormone production. This mechanism has been used to explain the elevated levels of estrogens in tamoxifen-treated premenopausal patients (Ravdin *et al*. 1988, Jordan *et al*. 1991). The aromatase enzyme, that converts androgens to estrogens, can also be elevated in estrogen-deprived cells adaptively *in vitro* (Yue *et al*. 2003) and can be stimulated through stromal cells that express prostaglandin E2, IL-6, 11 and tumor necrosis factor α (TNFα) (Schrey & Patel 1995). Indeed, it was shown that breast tumor tissues have higher levels of aromatase expression than peritumoral tissues (Bulun *et al*. 1996).

In recent years, antihormone resistance was also linked to the expression of membrane-associated ERs. The first membrane-associated ER that was identified was GPR30. It was demonstrated that the translocation of GPR30 to the cell surface significantly increased after estrogen treatment in tamoxifen-resistant breast cancer cells and its activity was mediated through the EGFR (Ignatov *et al*. 2010, Mo *et al*. 2013) and it is able to attenuate the inhibition of MAPK as well (Mo *et al*. 2013). It was also shown that GPR30 is able to upregulate aromatase expression in tamoxifen-resistant breast cancer cells, which can be linked to the sensitivity to AIs in breast cancer patients with acquired or *de novo* resistance to tamoxifen (Catalano *et al*. 2014). Another novel variant of ER that was recently identified is the membrane-bound ER-α36 that is associated with tamoxifen resistance *in vitro* (Wang & Yin 2015, Gu *et al*. 2017). However, the clinical roles these findings are yet to be determined.

### Ligand-independent and receptor-dependent mechanisms of resistance

Just like in the case with CRPC, one of the ligand-independent receptor-positive mechanisms of resistance in breast cancer could the activating mutations of the ER. Mutations of the ESR1 gene, that encodes the ER, have been identified in the LBD of the receptor in 14–54% of clinical samples from metastatic breast cancer patients and have also been linked to antihormone resistance (Robinson *et al*. 2013, Jeselsohn *et al*. 2014). These mutations are found most often in the metastases rather than in the primary tumors. Most mutations occur in positions Y537 and D538 and are described as gain-of-function mutations, which lead to constitutive ligand-independent activation of the ER (Robinson *et al*. 2013, Toy *et al*. 2013). Amino acid residues 537 and 538 are positioned in the AF-2 motif of the ER LBD so that mutations of these residues can induce a ligand-independent agonistic conformation of H12, closing the unoccupied LBD by interacting with residue at position 351 (Jordan *et al*. 2015). The ligand-free ER then recruits coactivators and activates the ER, even with the binding of tamoxifen (ligand dependent) (Nettles *et al*. 2008, Jordan *et al*. 2015, Fanning *et al*. 2016). We have also performed molecular dynamics modeling to demonstrate the conformational perturbation of the ER LBD with D538 mutation (Fanning *et al*. 2016) (Fig. 4).

ER-positive resistance in breast cancer is also attributed to the activation of growth factor pathways, such as HER2, IGF-1R and FGFR and stress-related kinases, such as AKT, JNK, MAPKs, c-SRC and others, that regulate posttranslational modifications of the ER and its coactivators that increase the receptor activity (Schiff *et al*. 2004, Shou *et al*. 2004, Santen *et al*. 2009*b*, Theoret *et al*. 2011). There is clinical evidence that proves that differential expression of various growth factor receptors in tamoxifen-resistant tumors are associated with resistance to tamoxifen and can play a role of a predictive clinical marker for therapy efficacy (Busch *et al*. 2015, Tomiguchi *et al*. 2016). Interestingly, these mechanisms can increase the membrane-associated ER activity with 17β-estradiol (E_2_) or even tamoxifen, also contributing to resistance in breast cancer. Increased levels of NF-κB and AP-1 can tether more ER to certain gene promoters and promote hormonal resistance (Zhou *et al*. 2007).

### Bypass pathway

Ligand and ER-independent mechanism depends upon MYC, Cyclin E1 and D1, p21 and p27 can promote progression through cell cycle despite tamoxifen therapy (Span *et al*. 2003, Butt *et al*. 2005, Perez-Tenorio *et al*. 2006, Chu *et al*. 2008). Antiapoptotic molecules, such as Bcl-xL, can be overexpressed to inhibit pro-apoptotic molecules and promote survival (Riggins *et al*. 2005).

## Evolution of acquired resistance in prostate and breast cancers

**Figure 5:**
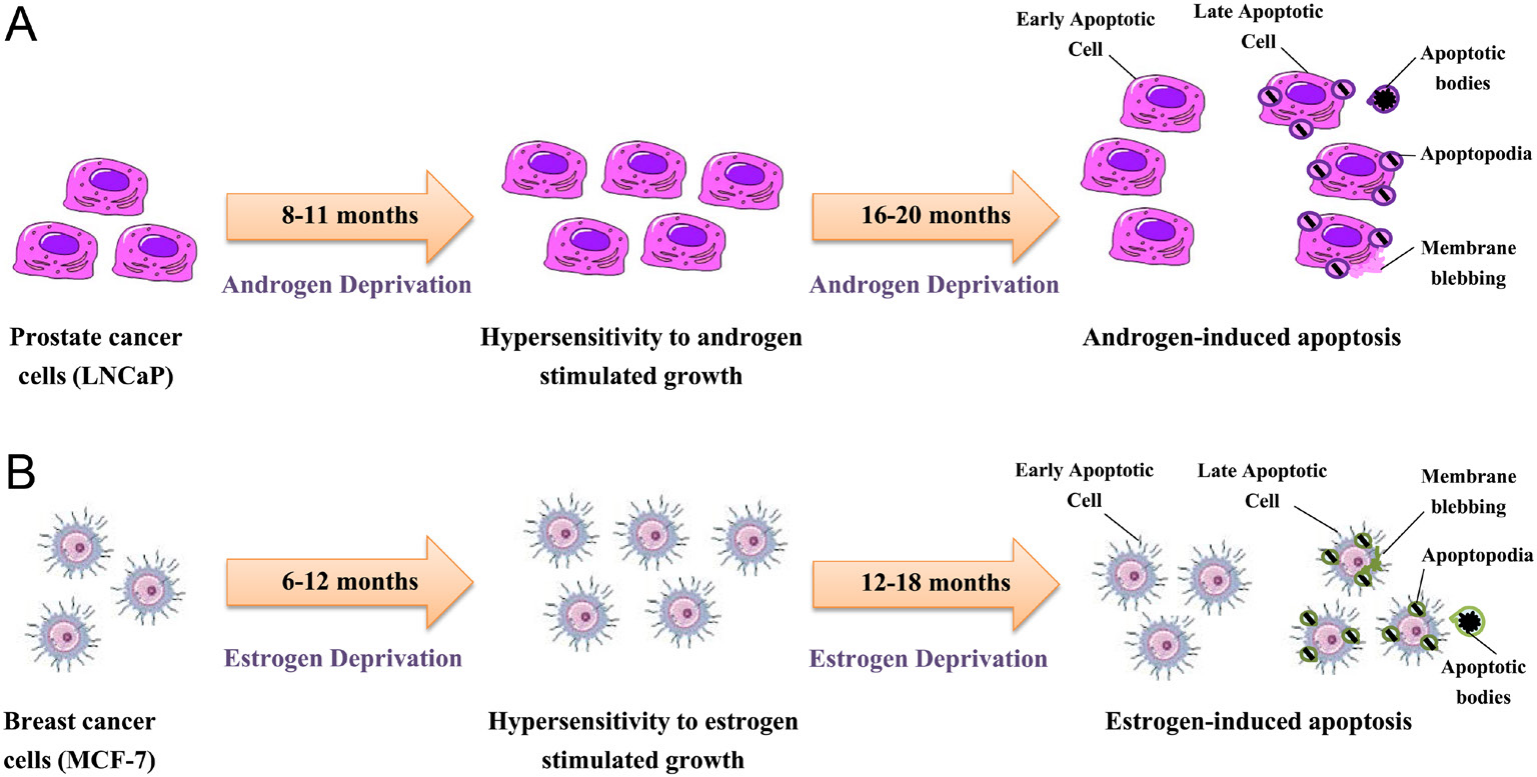
A schematic representation of the parallel cellular evolution of acquired hormone resistance to hormone deprivation in prostate and breast cancer cell models *in vitro*. (A) LNCaP cell line is an androgen-sensitive human prostate adenocarcinoma cell line. When LNCaP cells are cultured in an androgen depleted environment for 8–11 months *in vitro*, they become hypersensitive to androgen; and subsequently proliferate. With extended androgen depletion of 16–20 months, selection pressure occurs and LNCaP cells become vulnerable to androgens with death through apoptosis. Cells then exhibit the characteristic morphology of apoptosis with apoptotic membrane blebbing, followed by formation of membrane protrusions (apoptopodia, microtubule spikes, and beaded apoptopodia, beads-on-a-string appearance), ending with cellular fragmentation into apoptotic bodies. (B) MCF-7 cell line is an estrogen-sensitive human breast adenocarcinoma cell line. When MCF-7 cells are cultured in estrogen depleted environment for 6–12 months *in vitro*, they become hypersensitive to estrogen; and subsequently proliferate. With extended estrogen depletion of 12–18 months, selection pressure occurs and MCF-7 cells are now vulnerable to estrogens with death through apoptosis. Cells then exhibit the characteristic morphology of apoptosis.

Antihormonal therapy is standard for the treatment of recurrent and metastatic prostate cancer, however, up to 90% of these patients will ultimately fail and develop CRPC disease within 12–33 months after ADT. To understand this process of acquired resistance, numerous studies *in vitro* and *in vivo* were performed to simulate long-term ADT in prostate cancer cells to decipher the evolving mechanisms of acquired resistance (Kokontis *et al*. 1994, Umekita *et al*. 1996, Joly-Pharaboz *et al*. 2000) (Fig. 5). The studies to mimic long-term antiandrogen therapy in prostate cancer were performed using LNCaP cells, a popular AR-positive human cell line. Initial studies used various durations of steroid deprivation, with culture media containing charcoal-treated serum (Kokontis *et al*. 1994). Continuous passaging of cells in androgen-deprived conditions led to the selection of clones hypersensitive to androgens. However, longer androgen starvation (2 years) of these clones let to the isolation of cells that grow independently from androgen, with an unanticipated vulnerability (Kokontis *et al*. 1994). Low doses of androgen reduced the number of viable cells after 6 days of treatment (Kokontis *et al*. 1994). The authors (Kokontis *et al*. 1994) also noted a high level of AR protein and mRNA in these resistant cells compared to wild-type LNCaP cells. Expression of PSA protein and mRNA increased when treated with an androgen. Experiments *in vivo* using the same cells showed that the wild-type LNCaP tumors grew well in mice with androgen treatment; however, the derived resistant cell line grew only in castrated mice and treatment with DHT caused regression of the tumors (Umekita *et al*. 1996). The authors (Umekita *et al*. 1996) used further androgen deprivation of resistant cells in their experiments *in vitro* to derive a cell line that grew in androgen-deprived conditions as well as the wild-type cell line under testosterone stimulation. Interestingly, these resistant tumors were stimulated to grow with E_2_ and medroxyprogesterone acetate (MPA) and 5α-reductase inhibitor finasteride was able to partially reverse the tumoricidal actions of testosterone (Umekita *et al*. 1996).

Another group has performed similar *in vitro* and *in vivo* studies with long-term androgen-deprived LNCaP cells (Joly-Pharaboz *et al*. 2000). Wild-type LNCaP cells were passaged in culture medium supplemented with charcoal-treated serum for 1 year (Joly-Pharaboz *et al*. 2000). The resulting cell line grew independently from androgen, however, treatment with various androgens, and even E_2_ resulted in retarded cell growth due to apoptosis (Joly-Pharaboz *et al*. 2000). Experiments *in vivo* showed that androgen induced apoptosis and tumor regression with this model (Joly-Pharaboz *et al*. 2000).

Liang and coworkers (Chuu *et al*. 2011*b*) used variants of LNCaP prostate cancer cell lines to demonstrate that antiandrogen-resistant LNCaP cell lines with an AR-rich phenotype have a G1 cell cycle blockade in the presence of androgens by regulating cMyc, Skp2 and p27^kip^ via the AR. Additionally, they found that higher dosages of testosterone lead to more growth inhibition of relapsed tumors suggesting that the manipulation of androgen/AR signaling pathway may be a potential therapeutic target in AR-positive metastatic prostate cancer. Kawata and coworkers (Kawata *et al*. 2010) reported that prolonged treatment of a bicalutamide-resistant subline (LNCaP-BC2) with bicalutamide induces AR overexpression and androgen hypersensitivity to low levels of androgen. The authors identified the phosphorylated AR (pAR^210^) overexpression and a possible mechanism for androgen hypersensitivity. However, after long-term androgen deprivation, LNCaP prostate cancer cells evolve to be a cell population vulnerable to androgen-induced apoptosis (Chuu *et al*. 2011*a*). Nevertheless, continuous treatment with androgens eventually selects for cells that will be resistant to the apoptotic actions of androgens and grow. The authors speculated that it would be possible to use intermittent androgen deprivation (IAD) to slow the progression of resistance and use androgen therapy during the relapse after the ADT cycle to further control the tumor progression (Chuu *et al*. 2011*a*).

Clinically, there is evidence to support androgen-induced apoptosis in CRPC. Bruchovsky and coworkers (Akakura *et al*. 1993, Bruchovsky *et al*. 2000) used IAD to demonstrate that androgen action would inhibit growth of antiandrogen-resistant prostate cancer. There is evidence that IAD is able to prolong progression of resistant disease, and testosterone restoration between ablation therapy cycles can induce tumor regression in the laboratory *in vivo* (Sato *et al*. 1996) and in the clinic (Pether *et al*. 2003, Mathew 2008). In a recent viewpoint by Klotz and Higano (Klotz & Higano 2016), IAD was described as a viable alternative to the continuous androgen deprivation (CAD) in men with no underlying cardiovascular diseases. The IAD strategy was preferable with improved quality of life, cheaper health care costs, despite no observed advantage over CAD in terms of OS. Recently, Schweizer and coworkers (Schweizer *et al*. 2015) found a 50% response rate to androgen therapy when monitoring either PSA levels or radiologically identified CRPC disease.

Similar advances were made in studies of the antiestrogen resistance in breast cancer, and the evolution of breast cancer cells in the estrogen-free environment (Fig. 5). The evolution of MCF-7 breast cancer cells in estrogen deprivation conditions is similar to the evolution of LNCaP cells in response to androgen deprivation (Jordan *et al*. 2016).

Tamoxifen is a competitive inhibitor of estrogen action (Jordan 1984) and long-term adjuvant tamoxifen therapy was predicted to be essential to suppress breast tumor cell growth (Jordan 2014*b*). Early studies using MCF-7 breast cancer cell line transplanted into oophorectomized athymic mice demonstrated that although tumors eventually developed despite tamoxifen therapy (Osborne *et al*. 1987), the tumors, in fact, grew because of tamoxifen therapy (Gottardis & Jordan 1988, Gottardis *et al*. 1989*a*,*b*). Tamoxifen-stimulated tumors were growth stimulated by either tamoxifen or physiologic estradiol. As a result, no estrogen treatment or treatment with a pure antiestrogen (Gottardis *et al*. 1989*a*,*b*) prevented tumor growth. Discovery of this biology of early acquired resistance to tamoxifen preceded the clinical finding that either an AI or the pure antiestrogen fulvestrant were appropriate second-line therapies after tamoxifen failure in MBC (Howell *et al*. 2002, Osborne *et al*. 2002). This unique form of acquired resistance has clinical relevance in SERM pharmacology with a withdrawal response in MBC to SERMs tamoxifen and raloxifene (Howell *et al*. 1992, Dosik & Kaufman 2004, Lemmo 2016). The recent development (Fan *et al*. 2014*a*,*b*,*c*) of an *in vitro* model of acquired resistance to SERMs has provided important insight into how either tamoxifen (SERMs) or estrogen can stimulate tumor cell growth. Estrogen-stimulated growth in early acquired resistance to tamoxifen *in vivo* is via a genomic pathway, but with estrogen action at genomic sites blocked by tamoxifen. By contrast, tamoxifen stimulates tumor cell growth non-genomically by enhancing the IGFR1β pathway.

It is important to reemphasize that high-dose synthetic estrogen therapy was the first chemical therapy to treat any cancer (Haddow *et al*. 1944). However, Haddow (1970) noted that high-dose synthetic estrogen therapy was only effective at producing a 30% response rate in MBC 5 years following menopause. If estrogen was administered therapeutically nearer to the menopause then MCB grew. The reasons for this clinical observation were unknown and mechanisms were not deciphered during the 1950s–1970s, when high-dose estrogen was the standard of care for postmenopausal MBC. In the 1970s, tamoxifen, a non-steroidal antiestrogen (Jordan 2003), became the standard of care for all stages of breast cancer until the introduction of AIs in the late 1990s. There was no interest in understanding how high-dose estrogen therapy killed breast cancer cells despite the fact that high-dose DES produced a survival advantage over tamoxifen in a small trial in MBC (Ingle *et al*. 1981, Peethambaram *et al*. 1999).

It is therefore ironic that the study of acquired resistance to tamoxifen treatment in breast cancer should expose a vulnerability of antihormone-resistant breast cancer i.e.: estrogen-induced apoptosis (Wolf & Jordan 1993, Yao *et al*. 2000). Most importantly, the MCF-7 breast tumors developed acquired resistance to tamoxifen by cell selection over a 5-year period. Within two years, acquired resistance is evidenced by tamoxifen-stimulated growth and estrogen-stimulated growth; the growth stimuli are interchangeable. However, between 3 and 5 years of tamoxifen exposure, tamoxifen stimulates tumor growth but physiologic estrogen causes complete regression of small (<0.3 cm) tumors. The MCF-7 tumors rapidly regressed in response to E_2_. It was proposed (Yao *et al*. 2000) that estrogen treatment of recurrent breast cancer following the failure of long-term tamoxifen treatment, will result in a tumor regression and breast cancer cells will regain their responsiveness to estrogen for growth. Tamoxifen causes a decrease in mortality and prevents disease recurrence after 5 years of stopping the therapy, i.e., does not cause a rebound effect anticipated for a competitive inhibitor of estrogen action. The reason suggested is that a woman’s own estrogen causes estrogen-induced apoptosis in populations of vulnerable micrometastases that has long-term acquired resistance (Yao *et al*. 2000). This hypothesis in now supported by considerable clinical evidence reviewed elsewhere (Jordan 2014*a*, 2015*a*).

Song and coworkers (Song *et al*. 2001) reported that long-term estrogen deprivation leads to estrogen-induced apoptosis in LTED breast cancer cell population *in vitro*. Estrogen deprivation for a short time causes an elevation in the ER protein levels (Katzenellenbogen *et al*. 1987, Welshons & Jordan 1987). After 8–11 months of estrogen deprivation, MCF-7 cells acquire adaptive hypersensitivity to estrogen (Masamura *et al*. 1995), which is similar to LNCaP cells and hypersensitivity to androgen (Feldman & Feldman 2001). This may explain the early mechanism of AI resistance in breast cancer. Various cell models were developed over the years to study long-term estrogen deprivation in estrogen-free environment and using dilution cloning selection (Jiang *et al*. 1992, Pink *et al*. 1995, Song *et al*. 2001, Lewis *et al*. 2005*b*). Two breast cancer cell lines were selected after long-term estrogen deprivation (2 years). MCF-7:5C and MCF-7:2A cell lines were at first characterized as ER positive and non-responsive to estrogens or antiestrogens (Jiang *et al*. 1992, Pink *et al*. 1995); however, optimization of culture conditions dramatically altered these characteristics (Lewis *et al*. 2005*b*). The MCF-7:5C cells were shown to undergo low-concentration estrogen-induced apoptosis within a week of treatment in a concentration-dependent manner (Lewis *et al*. 2005*b*), and the intrinsic mechanism of estrogen-induced apoptosis was described (Lewis *et al*. 2005*a*, Fan *et al*. 2012, 2015). The MCF-7:2A cells undergo slow apoptotic alterations that occur within two weeks of treatment with estrogen. Both of these cell lines were used to investigate genome-wide alterations in estrogen-regulated gene expression profile involved in apoptosis (Ariazi *et al*. 2011).

## Current therapies for hormone-resistant prostate and breast cancers

Resistance to antihormonal therapy occurs in prostate and breast cancers, as new cell populations are selected after long-term sex steroid deprivation. These cells are characterized by sex hormone-independent growth.

It is believed that the AR in CRPC is still functional and can be abrogated to stop disease progression. Cytotoxic chemotherapy was routinely utilized to treat aggressive disease in the absence of targeted alternatives for CRPC prostate cancer. De Bono and coworkers (De Bono *et al*. 2010) compared cabazitaxel with the topoisomerase type II inhibitor mitoxantrone in mCRPC patients previously treated with docetaxel. Mortality was significantly decreased in the cabazitaxel group (De Bono *et al*. 2010). Smith and coworkers (Smith *et al*. 2013) evaluated cabozantinib (XL184), which is an orally bioavailable tyrosine kinase inhibitor that acts against MET and vascular endothelial growth factor receptor 2 (VEGFR2), in CRPC patients. They concluded that cabozantinib has clinical efficacy in CRPC improving PFS with a decrease of soft tissue lesions, resolution of bone scans, decline of bone turnover markers, pain and use of narcotic painkillers. However, the major strategic advance for the treatment of CRPC is the realization that the AR is still functional in CRPC and, like in breast cancer, remains a potential target.

New antihormonal agents are improving the prognosis of CRPC. Abiraterone acetate (Barrie *et al*. 1994) is an inhibitor of cytochrome P450 (CYP17) (Fig. 1), which plays an essential role in *de novo* intratumoral androgen production from cholesterol in CRPC tumors (Locke *et al*. 2008). This therapeutic approach to treat prostate cancer is analogous to the use of adjuvant therapy with AIs in postmenopausal breast cancer patients (Fig. 3). De Bono and coworkers (de Bono *et al*. 2011) evaluated abiraterone acetate in patients with mCRPC who have received chemotherapy and demonstrated that the inhibition of androgen biosynthesis by abiraterone prolonged the OS. Other approaches target the AR with new antiandrogens.

Scher and coworkers (Scher *et al*. 2010) evaluated the antitumor activity and safety of enzalutamide, which blocks AR activity in men with CRPC (Fig. 2). Increasing doses of enzalutamide reduced serum PSA and stabilized bone disease in 56% of patients (Scher *et al*. 2010). Recently, Penson and coworkers (Penson *et al*. 2016) compared the efficacy of enzalutamide and bicalutamide in CRPC. Enzalutamide decreased the mortality of patients by 76% with a median PFS of 19.4 months compared to bicalutamide with a median PFS of 5.7 months. There was a significant increase in PFS with enzalutamide in the proportion of patients with a ≥50% PSA response, time to PSA progression and radiographic PFS in metastatic patients. Advantages of enzalutamide were observed in both metastatic and nonmetastatic subgroups. However, evidence is emerging on acquired resistance to abiraterone and enzalutamide (Attard & Antonarakis 2016, Bubley & Balk 2017, Gupta *et al*. 2017).

Several new antiandrogens are in early clinical development. The antiandrogen ARN-509 developed by Janssen Research & Development is an example of a potent competitive pure antiandrogen that has been evaluated in phase I/II trials in CRPC patients. In phase I trial, ARN-509 was well tolerated with fatigue being the most reported side effect (Rathkopf *et al*. 2013). In the phase II study, ARN-509 demonstrated an 80–90% efficacy in patients with naïve CRPC in both metastatic and nonmetastatic settings. There was a 29% response rate in mCRPC patients previously treated with abiraterone, reducing the PSA levels by more than 50% (Rathkopf *et al*. 2012). The novel small peptide EPI-001 targets the N-terminal domain of the AR containing the activating function-1 region (AF-1). This interrupts the AR’s interaction with other proteins and androgen response elements in the androgen-responsive genes promoters. As a result, transcriptional activity is disrupted (Andersen *et al*. 2010). This peptide has not entered clinical trial, but showed promising results in the CRPC xenograft models (Andersen *et al*. 2010). A novel selective AR downregulating drug (SARD) AZD3514 had limited tolerability in CRPC patients in a phase I trial with modest antitumor activity; however, it did show activity in 17–25% of patients reducing PSA by more than 50% (Omlin *et al*. 2015). The authors concluded that developing SARDs in the future for treatment of CRPC may hold merit (Omlin *et al*. 2015).

Despite the use of long-term antiestrogen adjuvant therapy for breast cancer, approximately 50% of patients have disease recurrence. The question we must ultimately address is how we improve response rates? Though tamoxifen was approved initially for treatment of MBC in both pre- and postmenopausal women, AIs became the first-line therapy for postmenopausal breast cancer patients who did not have any prior hormonal therapy or have recurred within 12 months after previous adjuvant AI therapy. However, if the tumors recur in less than 12 months after hormonal therapy with an AI, then tamoxifen is recommended or a pure antiestrogen fulvestrant as second-line therapies. Recently, Robertson and an international team of colleagues (Robertson *et al*. 2016) in a phase III clinical trial have demonstrated superiority of fulvestrant over anastrazole as first-line therapy in postmenopausal patients with metastatic of locally advanced breast cancer. For premenopausal women, tamoxifen can be prescribed as first-line adjuvant hormonal therapy and AIs or fulvestrant can be used as second- and third-line therapies in case of cancer recurrence, but only with ovarian function suppression (Abderrahman & Jordan 2016). Antihormone resistance eventually occurs after exhaustive antihormone therapy fails. However, depending on the size and location of the metastasis cytotoxic chemotherapy is more likely to be used after a failed AI therapy rather than second or third-line antihormone agents.

New strategies for the treatment of hormone-refractory breast cancer are evolving based on inhibition of aberrant pathways. Abnormalities in the CDK4/6 and the mTOR pathways play a crucial role in the pathogenesis of breast cancer. These pathways are therapeutic targets for the treatment of naïve MBC or antihormone-resistant breast cancer. In phase I/II clinical studies (Schwartz *et al*. 2011), palbociclib, which is a specific CDK4/6 inhibitor (O’Leary *et al*. 2016), demonstrated an excellent bioavailability, mild to moderate adverse effects, and a well-tolerated toxicity. In phase III clinical study called PALbociclib Ongoing trials in the Management of breast cAncer-3 (PALOMA-3) (Turner *et al*. 2015) the combination of palbociclib with endocrine therapy significantly improves PFS. All these data resulted in palbociclib receiving an FDA approval in 2015 as a first-line treatment for advanced postmenopausal ER-positive/HER2-negative breast cancer in combination with letrozole.

Numeral studies with mTOR inhibitors (i.e. everolimus, temsirolimus, deforolimus) show promise in the ER-positive and/or HER2-positive breast cancer (Fasolo & Sessa 2008, Vicier *et al*. 2014, Baselga *et al*. 2017). The combination of everolimus with either an AI (Beck *et al*. 2014, Finn *et al*. 2015) or fulvestrant (Beaver & Park 2012, Sun *et al*. 2016, Pritchard *et al*. 2017) demonstrated clinical efficacy. The Breast Cancer Trials of OraL EveROlimus-2 (BOLERO-2) (Baselga *et al*. 2012), combined everolimus and exemestane for women with advanced ER-positive/HER2-negative breast cancer who previously failed AI therapy. In BOLERO-2, everolimus improved PFS in trastuzumab-resistant patients. Interestingly, in an early study with an mTOR inhibitor (deGraffenried *et al*. 2004) rapamycin ester (CCI-779) treatment restored tamoxifen response in tamoxifen-resistant breast carcinoma (Yu *et al*. 2001).

Regrettably, combination therapies with CDK4/6 inhibitors or mTOR inhibitors with an antihormonal therapy do not result in lives saved, although life extension is a positive benefit. The question now becomes: how can adjuvant endocrine therapy be advanced based on what we now know from current clinical trials? There is a linear progression from therapeutic success in MBC to trials of adjuvant therapy, but we suggest this may not be that simple with CDK4/6 inhibitors and mTOR inhibitors.

The high monthly cost for both CDK4/6 inhibitors and mTOR inhibitors (Carey & Perou 2015), and the toxicity profile of grade 3/4 side effects with palbociclib (Finn *et al*. 2015) (i.e. neutropenia, leukopenia, and lymphopenia), and grade 1/2 side effects with everolimus (Baselga *et al*. 2012) (i.e. fatigue, stomatitis, anorexia, diarrhea, noninfectious pneumonitis, metabolic disorders with hyperglycemia and hematologic disorders) hinder their utilization as a useful long-term adjuvant treatment. These systemic side effects and financial costs will reduce patient compliance and the value of antihormone therapy will be lost. It is difficult to maintain compliance for current antihormonal agents for 5 years, so an increase in side effects will result in the failure to control disease recurrence. We suggest another path in the final sections of this review.

## Consideration for implementing a pathway forward that saves lives following a diagnosis of prostate and breast cancer

Enormous progress has occurred in the last 40 years in the approach to treating prostate and breast cancer. In the period 1967–1977 there were no proactive detection programs, diagnosis was usually late stage disease and the word cancer was not used. Quite rightly, cancer had the reputation as a death sentence. Radical surgery and radiotherapy were the major weapons in the physicians armamentarium and chemical therapy (chemotherapy) was primitive. Medical oncology was an emerging specialty. High-dose estrogen therapy was effective in 30% of both metastatic breast and prostate cancers, but this was a paradox as both breast and prostate cancers were known to be sex steroid dependent. Mechanisms were unknown.

A significant step forward occurred in breast cancer treatment with the publication of a symposium at King’s College, Cambridge (28–29th September, 1977) in the October Supplement of Reviews in Endocrine-Related Cancer (Jordan 1978), the fore-runner of the current Society for Endocrinology journal Endocrine-Related Cancer. The conclusions, which hold true today, were: (1) treating animals with a large tumor burden cannot affect a cure; (2) the tumor ER is important to predict a response to tamoxifen; (3) treating with tamoxifen early in tumorigenesis: i.e. low tumor burden, produces some protection for animals; (4) longer treatment with tamoxifen is superior to short treatment in animals with microscopic disease.

This and subsequent publications (Jordan 1978, Jordan *et al*. 1979, 1980, Jordan & Allen 1980) triggered the move to long-term adjuvant antiestrogen therapy proven to save lives (Goss *et al*. 2005, Davies *et al*. 2013). As illustrated in this current review of prostate and breast cancer treatments, the diseases run different courses. Adjuvant therapy in prostate cancer is not implemented in the same way as is routine for breast cancer. In breast cancer, antihormone therapy is used to benefit patients in all stages of breast cancer, but the same is not true for prostate cancer. ADT is only used in MPC, locally advanced or recurring cases. Nevertheless, our review illustrates that the evolution of acquired resistance for both breast and prostate cancer is similar. Mechanisms of acquired resistance are broadly the same or the adaptions of alternate growth stimulating pathways are similar. The major risk factor for both prostate and breast cancer, is age. A primary consideration is to seek effective therapeutic solutions for our aging population. Resources are scarce and our goal of achieving chemoprevention of breast and prostate cancers has fallen short. We still do not know precisely who will develop breast or prostate cancer, and why. Treating large population to benefit a few, who do not know their disease was prevented, was an ineffective approach. Side effects from any chemopreventive intervention are unacceptable to any but the most committed high-risk woman who wishes to prevent breast cancer. A strategy to prevent prostate cancer using an inhibitor of 5α-reductase was scientifically sound (Homma *et al*. 1997, Andriole *et al*. 2004, Thorpe *et al*. 2007) but outcomes were controversial due to potential risks of high-grade prostate cancers and this advance in health care was abandoned (FDA 2011, Theoret *et al*. 2011). The chemoprevention solution has overwhelmed healthcare systems. There is neither physician time to address individual needs for chemoprevention (Smith *et al*. 2017) nor, it seems, physician knowledge about options (Smith *et al*. 2017). We must, therefore, do what can be done to aid patients with breast and prostate cancers. This strategy must be inexpensive, globally applicable and aim to keep as many individuals well who can continue to contribute effectively to the welfare of the family. This essential goal will impact on the welfare of countries as each family unit can contribute to the economy of that country. In the final section we will address what can be done, how and why the approach is feasible.

## An approach to global health care maintenance in prostate and breast cancers

Tamoxifen has taught us the fundamental laws of clinical therapeutics. To this day, antihormone therapy of MBC plus/minus chemotherapy or precision medicines (to block cell replication or the survival pathways that subvert antihormone action away from the ER growth pathway) can delay but not prevent death (Abderrahman & Jordan 2016). The same medicine tamoxifen or now an AI (letrozole) applied as a long-term adjuvant therapy, can delay recurrence and decrease mortality. Laboratory studies of acquired resistance to antihormone therapies (Wolf & Jordan 1993, Yao *et al*. 2000, Song *et al*. 2001) opened the door to understanding the ‘carry over’ effect of long-term adjuvant antiestrogen therapy that decreases mortality after adjuvant therapy is stopped (Fisher *et al*. 2005, Cuzick *et al*. 2007, Powles *et al*. 2007).

The knowledge of mechanisms in adjuvant therapy in breast cancer can now be built upon to enhance survivorship and improve the quality of life during long-term adjuvant therapy. By contrast, the urologic community must decide whether select patients, destined to remain hormone responsive, could or should be treated with adjuvant ADT. An approach would be to correlate the genomics of indolent primary tumors with outcomes at recurrence that is antihormone responsive MPC. In this way, analysis of large data sets could save lives. The identification of those tumors that recur with MPC but subsequently respond to ADT would be candidates for adjuvant approaches in the future. Indeed, long drug holidays or androgen therapy may benefit patients with androgen-induced apoptosis of microscopic disease. Until that time, the strategy of long-term adjuvant control of prostate cancer cannot be considered.

For breast cancer, by contrast, the landscape holds numerous affordable possibilities. The AIs have reduced RR, with fewer serious side effects, but results of survivorship are less clear than with the SERM tamoxifen. However, the creation of the ‘estrogen-free’ woman for the remainder of her life, during adjuvant AI therapy, has concerns for general health. Osteoporosis is a concern, as is the less well-defined issues of coronary heart disease (CHD) and reduced mental capacity. This may include exacerbation of Alzheimer’s disease for our aging population. Clearly, large populations of patients with Alzheimer’s should be examined to determine whether breast cancer adjuvant treatment with either tamoxifen or AIs advance Alzheimer’s onset or exacerbates symptoms and severity.

The ‘SERMs Solution’ (Lerner & Jordan 1990) for the chemoprevention of breast cancer now has a role to improve long-term adjuvant therapy. The original proposal for SERM was:

‘Important clues have been garnered about the effects of tamoxifen on bone and lipids so it is possible that derivatives could find targeted applications to retard osteoporosis or atherosclerosis. The ubiquitous application of novel compounds to prevent diseases associated with the progressive changes after menopause may, as a side effect, significantly retard the development of breast cancer.’ (Lerner & Jordan 1990)

Following the success of the pioneering SERM tamoxifen, the medicinal chemistry community has advanced numerous safe and widely used new SERMs including raloxifene, bazedoxifene and ospemifene (Maximov *et al*. 2013). All are FDA approved for different indications in postmenopausal women’s health. Only raloxifene has a cancer indication; the chemoprevention of breast cancer in high-risk postmenopausal women. Lasofoxifene is not yet approved but promises not only to reduce fracture risk in osteoporosis, reduce breast cancer incidence, and reduce strokes, but is the only SERM proven to reduce CHD (Cummings *et al*. 2010). Turning around the ‘SERM solution’ for women’s health one more time, there is a strategic opportunity for medicinal chemists to solve one of the important molecular mechanisms of acquired AI resistance, i.e.: expansion and mutations of the ER. However, this must be achieved not with an orally active pure antiestrogen (Abderrahman & Jordan 2016), but a SERM that destroys the ER.

Orally active ‘pure antiestrogens’ are a current focus of medicinal chemistry with the goal of being effective therapies in MBC after the failure of AI therapy (Abderrahman & Jordan 2016). But this is not good enough. The oral pure antiestrogen solution as a future adjuvant therapy would still keep women estrogen free.

Medicinal chemists already know how to make a SERM that maintains bone density in ovarierectomized rats, but destroys the tumor cell ER (Willson *et al*. 1994, Bentrem *et al*. 2001). The compound GW-5638 (Etacstil), was reported 20 years ago! The acrylic ‘antiestrogenic’ side chain when attached to the triphenyethylene core, fits appropriately into the ER ligand-binding domain but causes perturbation of the ER complex, resulting in rapid destruction (Wu *et al*. 2005). This acrylic side chain is a recurrent feature of the ‘new pure antiestrogens’ under investigation (Abderrahman & Jordan 2016).

A new SERM that destroys tumor ER, used as an adjuvant therapy, would not only enhance survivorship by reducing recurrence noted with AIs, but also improve woman’s health. Current problems of compliance can be addressed and improved. Women struggle with poor quality of life with AIs. Even a 3 month trial of local estrogen (or testosterone) is currently being evaluated to eliminate vaginal atrophy (Melisko *et al*. 2017), but a SERM could also achieve the same result (Jordan 2017*b*). Quality of life and being well is an essential component of patient survival. Stopping long-term adjuvant therapy prematurely, because of a lack of compliance, is the same as deciding upon a couple of years of adjuvant therapy. To stop adjuvant therapy early is not recommended. Indeed, the value of more than 5 years of adjuvant therapy has been evaluated. Ten years of adjuvant tamoxifen is superior to 5 years of adjuvant tamoxifen in lives saved, but only in the five years after completion of 10 years of adjuvant tamoxifen (Davies *et al*. 2013). This is the essential role of estrogen-induced apoptosis, but the value in lives saved with an adjuvant AI is less clear (Goss *et al*. 2016).

**Figure 6:**
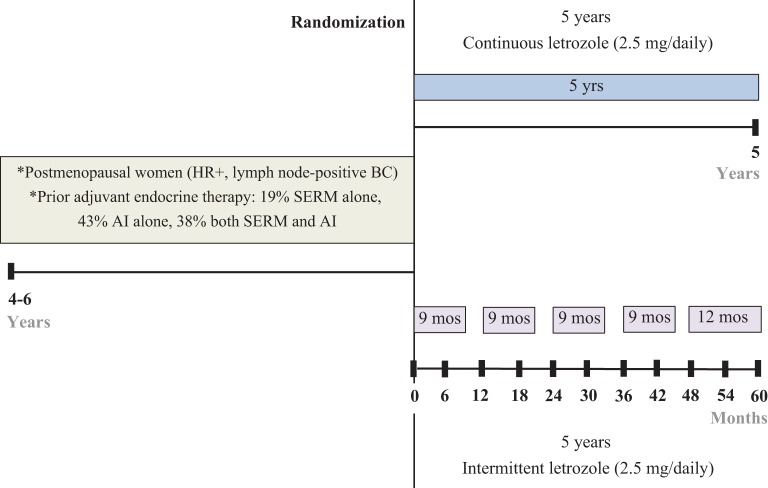
A schematic representation of the Study of Letrozole Extension (SOLE) trial. SOLE is a phase III randomized clinical trial of continuous vs intermittent letrozole in postmenopausal women who had received 4–6 years of adjuvant endocrine therapy for hormone receptor (HR)- positive, lymph node- positive, early-stage breast cancer (BC). The rationale of SOLE trial was to test if 3-month treatment-free intervals during extended adjuvant endocrine therapy, would improve disease-free survival (DFS). The underpinning of this hypothesis is based on the theory that letrozole withdrawal for 3 months would allow a degree of estrogenic stimulation toward residual resistant disease, and subsequently the residual disease would become susceptible to letrozole reintroduction. The primary endpoint was DFS (randomization until invasive local, regional, distant recurrence or contralateral BC; 2nd malignancy; death). Postmenopausal women with prior 4–6 years of adjuvant endocrine therapy, were randomized into two arms: first arm is control which is continuous letrozole of 2.5 mg/daily for 5 years, and the second arm is intermittent letrozole of 2.5 mg/daily for 9 months in the first 1–4 years and fully at year 5. The trial concluded no difference in DFS among the two arms but for the first time pre-planned medication non-adherence is not harmful. This can provide a treatment-side effects and financial relief to many patients.

The Study of Letrozole Extension (SOLE) addressed the issue of 3 month drug holidays annually (Fig. 6) but now is an opportunity to advance a new adjuvant therapy strategy. The goal of the study was to establish that a woman’s own estrogen would benefit patients by triggering estrogen-induced apoptosis. Invoking a physiologic antitumor mechanism would reduce micrometastatic disease and decrease recurrence. The hypothesis was based on published laboratory evidence (Wolf & Jordan 1993, Yao *et al*. 2000, Song *et al*. 2001). Though recommended at the time, administration of low-dose estrogen was considered too dangerous for patients without clinical evidence of efficacy and safety. The clinical studies have now occurred (Ellis *et al*. 2009, Anderson *et al*. 2012) so the laboratory concept is sound. The SOLE study is now reported (Colleoni *et al*. 2017) but shows no benefit for intentional 3 month annual drug holidays for 4 consecutive years of letrozole adjuvant therapy. Nevertheless, the SOLE trial provides significant important new information for two further advances in women’s health.

**Figure 7:**
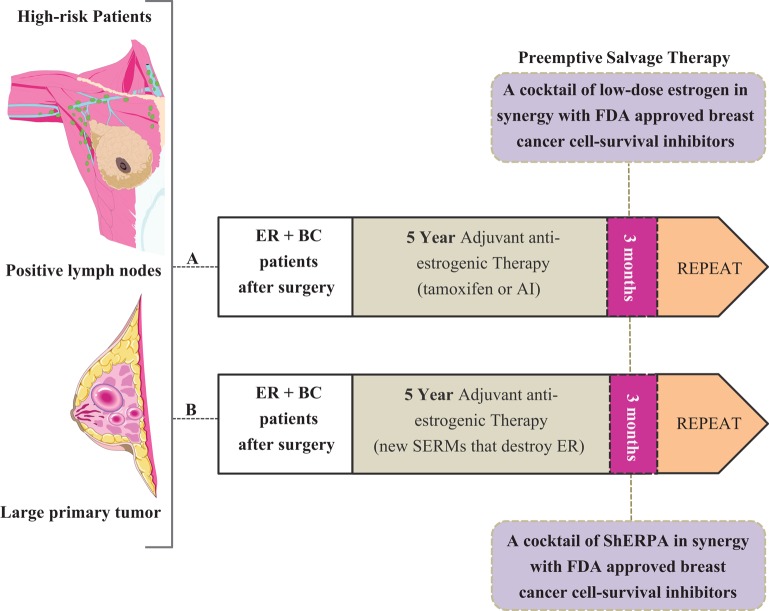
A schematic representation of the proposed design (alongside a proposed optimized version) for the preemptive salvage therapy. (A) Breast cancer patients who are ER- positive after surgery and at high risk of recurrence (this includes large primary tumors and positive lymph nodes at diagnosis), can harness the benefits of long-term estrogen deprivation, with a preemptive salvage therapy, aiming at clearing occult micrometastases. After 5 years of adjuvant antihormonal therapy with either tamoxifen or AIs, breast cancer cell populations undergo selection pressure. The new long-term estrogen-deprived (LTED) breast cancer cell populations are now vulnerable to a woman’s own estrogen through apoptosis (aka estrogen-independent). Whereas, they would normally grow with estrogen within 5 years past menopause (aka estrogen-dependent). The clinically observed response rate to low-dose estrogen therapy was 30% in metastatic breast cancer. Estrogen can act in synergy with other FDA approved breast cancer cell survival inhibitors or apoptosis promoters. This synergy can potentially increase the response rate above 30%. (B) Panel A can be optimized. Estrogen deprivation can be achieved with a new SERM that degrades the ER, preventing future drug resistance and receptor mutations. As one example, there is an orally active SERD, GW5638, which is metabolically hydroxylated to GW7604, in the same way, tamoxifen is metabolically activated to 4-hydroxytamoxifen. Unlike tamoxifen, GW7608 triggers the destruction of ER in BC cells, while retaining an estrogenic tickle at ER elsewhere (i.e. bones and serum lipids). Although GW7608 is a SERD for degrading the ER, it is also a SERM due to its agonistic and antagonistic mechanism of action at different tissue levels. A similar mechanistic SERM/SERD compound can improve estrogen deprivation (with an AI) by destroying the ER, while maintaining women’s health. In addition, estrogen in the proposed 3-month drug holiday can be replaced with selective human estrogen receptor partial agonist (ShERPA). These compounds mimic estrogen without causing significant uterine growth and were found to inhibit the growth of endocrine-independent tamoxifen-resistant breast cancer cell lines.

Our goal here is to propose a long-term therapeutic strategy that not only builds on past clinical experience, but also introduces a new strategic concept for adjuvant therapy to improve patient care globally. The new information from the SOLE trial is a first step forward. Firstly, the fact that a patient can stop therapy for 3 months and then restart adjuvant therapy allows compliance issues, due to side effects, to be addressed. A vigilant breast team can now offer returning to continuous adjuvant AI therapy to patients in distress. Secondly, the 3 month adjuvant window can now be used to create an advance in adjuvant therapy to reduce the micrometastatic tumor burden for those patients known to be at high risk for recurrence and death if a second five years of adjuvant therapy is not enforced (Abderrahman & Jordan 2017). This is important, as a short-term intensive preemptive salvage therapy (Fig. 7), because cost and toxic side effects for current precision medicines (palbociclib and everolimus) will make years of combination therapy with an innovative antiestrogen therapy impractical (Carey & Perou 2015). But how should the clinical community advance the new therapeutic innovations?

Firstly, there has to be clearly defined patient population that is at high risk of recurrence despite long-term adjuvant antihormone therapy. Recent data reported by the Early Breast Cancer Trialists Collaborative Group (EBCTCG) (Pan *et al*. 2017) define that high-risk population that recurs following 5 years of adjuvant tamoxifen. Follow-up is for 15 years and, not surprisingly, RR depend upon the size of the original primary tumor and the number of axillary lymph nodes. Secondly, there needs to be a defined combination of physiologic estrogen plus a cocktail of precision medicines to reduce the burden of micrometastatic disease and avoid recurrence (Jordan *et al*. 2016). The goal is to kill micrometastatic disease not just hold cancer cell growth. Understandable concerns are raised still about the safety of low-dose estrogen (Reeder-Hayes & Muss 2017) and this is appropriate, but medicinal chemists are already addressing the problem. Raloxifene derivatives that trigger apoptosis in LTED breast cancer, have been reported (Xiong *et al*. 2016) and clinical trials are planned.

Secondly, studies using FDA approved low-dose estrogen (FDA approved!) and cocktails of precision survival inhibitors in LTED breast cancer can be confirmed to be effective in LTED MBC. The strategy would be in place for the evaluation of the best selective human estrogen receptor partial agonist (ShERPA) (Xiong *et al*. 2016) to then go into the adjuvant testing. Already liquid biopsies for breast cancer with low tumor burden are being advanced in clinical testing (Phallen *et al*. 2017). This technical advance will support adjuvant monitoring of apoptotic success.

Breast cancer treatment must evolve to improve women’s health. The view that ‘this is good enough’ must not take hold. A new SERM for adjuvant therapy must be the best to improve women’s health and the best to prevent breast cancer recurrences. Knowledge now exists for the eventual implementation of a new preemptive salvage therapy (Fig. 7) strategies based on the planned drug holidays in SOLE, and the use of a new SERM with a positive health pharmacology to replace AIs. Together, a new adjuvant SERM that destroys the ER with a SOLE adjuvant design of 3 month therapeutic windows, using precision medicines that kill micrometastatic breast cancer, would be an optimal strategy for breast cancer therapy to achieve. This would be cheap once patenting of precision medicines lapses, easy to administer orally and would address all current issues with current AI therapy. Nevertheless, it will be said that the plan could take decades so it cannot (should not) be attempted.

Forty years ago (Jordan 1978), there was no long-term adjuvant therapy, no understanding of dangerous side effects with tamoxifen, i.e. endometrial cancer, no AIs or SERMs for women’s health. The strategy of long-term adjuvant therapy in breast cancer was considered ‘mindless’ (Stoll 1991) at best and would not improve patient care, and dangerous at worst as it would encourage premature drug resistance. These medical opinions did not go unchallenged (Jordan 1991). Instead, the translational adjuvant strategies using antiestrogens conceived in the laboratory in the 1970/80s, gave patients fewer RR, unanticipated major decreases in mortality, fewer contralateral breast cancers, awareness of the link between tamoxifen and endometrial cancer, an understanding of the unique mechanisms of acquired resistance to antihormones that can be used to treat breast and prostate cancer and the new science of sex steroid-induced apoptosis. It is now prudent to plan for improvements in clinical care and build on past clinical advances.

## Declaration of interest

The authors declare that there is no conflict of interest that could be perceived as prejudicing the impartiality of this review.

## Funding

This work was supported by the National Institutes of Health NIH, MD Anderson’s Cancer Center support grant CA016672 and Susan G. Komen for the Cure Foundation under award number SAC100009, and Cancer Prevention Research Institute of Texas (CPRIT) for the STARs and STARs plus Awards. V C J thanks the benefactors of the Dallas/Ft. Worth Living Legend Chair of Cancer Research for their generous support. R C would like to thank the Romanian National Authority for Scientific Research and Innovation, Project no. 1.1.2/2017, CNCS – UEFISCDI, project number PN-II-RU-TE-2014-4-0422. Y H would like to thank the King Faisal Specialist Hospital & Research Centre (Gen. Org) (KFSH & RC-Jed) and the Royal Embassy of Saudi Arabia-Cultural Bureau in the USA for their financial support.

## References

[bib1] Abderrahman B Jordan VC 2016 Improving long-term adjuvant anti-oestrogenic therapy for breast cancer. Clinical Pharmacist. (10.1211/CP.2016.20201203)

[bib2] Abderrahman B Jordan VC 2017 Rethinking extended adjuvant antiestrogen therapy to increase survivorship in breast cancer. JAMA Oncology [epub]. (10.1001/jamaoncol.2017.351) 29145574

[bib3] Ahmann FR Citrin DL deHaan HA Guinan P Jordan VC Kreis W Scott M Trump DL 1987 Zoladex: a sustained-release, monthly luteinizing hormone-releasing hormone analogue for the treatment of advanced prostate cancer. Journal of Clinical Oncology 5 912–917. (10.1200/JCO.1987.5.6.912) 2953870

[bib4] Akakura K Bruchovsky N Goldenberg SL Rennie PS Buckley AR Sullivan LD 1993 Effects of intermittent androgen suppression on androgen-dependent tumors. Apoptosis and serum prostate-specific antigen. Cancer 71 2782–2790. (10.1002/1097-0142(19930501)71:9<2782::AID-CNCR2820710916>3.0.CO;2-Z) 7682149

[bib5] Albertsen PC Moore DF Shih W Lin Y Li H Lu-Yao GL 2011 Impact of comorbidity on survival among men with localized prostate cancer. Journal of Clinical Oncology 29 1335–1341. (10.1200/JCO.2010.31.2330) 21357791 PMC3084001

[bib6] Alkner S Bendahl PO Ehinger A Lovgren K Ryden L Ferno M 2016 Prior adjuvant tamoxifen treatment in breast cancer is linked to increased AIB1 and HER2 expression in metachronous contralateral breast cancer. PLoS ONE 11 e0150977 (10.1371/journal.pone.0150977) 26959415 PMC4784945

[bib7] Andersen RJ Mawji NR Wang J Wang G Haile S Myung JK Watt K Tam T Yang YC Banuelos CA 2010 Regression of castrate-recurrent prostate cancer by a small-molecule inhibitor of the amino-terminus domain of the androgen receptor. Cancer Cell 17 535–546. (10.1016/j.ccr.2010.04.027) 20541699

[bib8] Anderson KM Liao S 1968 Selective retention of dihydrotestosterone by prostatic nuclei. Nature 219 277–279. (10.1038/219277a0) 5671431

[bib9] Anderson WF Katki HA Rosenberg PS 2011 Incidence of breast cancer in the United States: current and future trends. Journal of the National Cancer Institute 103 1397–1402. (10.1093/jnci/djr257) 21753181 PMC3176776

[bib10] Anderson GL Chlebowski RT Aragaki AK Kuller LH Manson JE Gass M Bluhm E Connelly S Hubbell FA Lane D 2012 Conjugated equine oestrogen and breast cancer incidence and mortality in postmenopausal women with hysterectomy: extended follow-up of the Women’s Health Initiative randomised placebo-controlled trial. Lancet Oncology 13 476–486. (10.1016/S1470-2045(12)70075-X) 22401913 PMC3412626

[bib11] Andriole G Bostwick D Brawley O Gomella L Marberger M Tindall D Breed S Somerville M Rittmaster R Group RS 2004 Chemoprevention of prostate cancer in men at high risk: rationale and design of the reduction by dutasteride of prostate cancer events (REDUCE) trial. Journal of Urology 172 1314–1317. (10.1097/01.ju.0000139320.78673.2a) 15371831

[bib12] Ariazi EA Cunliffe HE Lewis-Wambi JS Slifker MJ Willis AL Ramos P Tapia C Kim HR Yerrum S Sharma CGN 2011 Estrogen induces apoptosis in estrogen deprivation-resistant breast cancer through stress responses as identified by global gene expression across time. PNAS 108 18879–18886. (10.1073/pnas.1115188108) 22011582 PMC3223472

[bib13] Arora VK Schenkein E Murali R Subudhi SK Wongvipat J Balbas MD Shah N Cai L Efstathiou E Logothetis C 2013 Glucocorticoid receptor confers resistance to antiandrogens by bypassing androgen receptor blockade. Cell 155 1309–1322. (10.1016/j.cell.2013.11.012) 24315100 PMC3932525

[bib14] Attard G Antonarakis ES 2016 Prostate cancer: AR aberrations and resistance to abiraterone or enzalutamide. Nature Reviews Urology 13 697–698. (10.1038/nrurol.2016.212) 27804988

[bib15] Attia DM Ederveen AG 2012 Opposing roles of ERalpha and ERbeta in the genesis and progression of adenocarcinoma in the rat ventral prostate. Prostate 72 1013–1022. (10.1002/pros.21507) 22025007

[bib16] Barboro P Salvi S Rubagotti A Boccardo S Spina B Truini M Carmignani G Introini C Ferrari N Boccardo F 2014 Prostate cancer: prognostic significance of the association of heterogeneous nuclear ribonucleoprotein K and androgen receptor expression. International Journal of Oncology 44 1589–1598. (10.3892/ijo.2014.2345) 24626777

[bib17] Barrie SE Potter GA Goddard PM Haynes BP Dowsett M Jarman M 1994 Pharmacology of novel steroidal inhibitors of cytochrome P450(17) alpha (17 alpha-hydroxylase/C17-20 lyase). Journal of Steroid Biochemistry and Molecular Biology 50 267–273. (10.1016/0960-0760(94)90131-7) 7918112

[bib18] Baselga J Campone M Piccart M Burris HA 3rd Rugo HS Sahmoud T Noguchi S Gnant M Pritchard KI Lebrun F 2012 Everolimus in postmenopausal hormone-receptor-positive advanced breast cancer. New England Journal of Medicine 366 520–529. (10.1056/NEJMoa1109653) 22149876 PMC5705195

[bib19] Baselga J Morales SM Awada A Blum JL Tan AR Ewertz M Cortes J Moy B Ruddy KJ Haddad T 2017 A phase II study of combined ridaforolimus and dalotuzumab compared with exemestane in patients with estrogen receptor-positive breast cancer. Breast Cancer Research and Treatment 163 535–544. (10.1007/s10549-017-4199-3) 28324268 PMC5448790

[bib20] Baum M Budzar AU Cuzick J Forbes J Houghton JH Klijn JG Sahmoud T Group AT 2002 Anastrozole alone or in combination with tamoxifen versus tamoxifen alone for adjuvant treatment of postmenopausal women with early breast cancer: first results of the ATAC randomised trial. Lancet 359 2131–2139. (10.1016/S0140-6736(02)09088-8) 12090977

[bib21] Beatson GT 1896 On the treatment of inoperable cases of carcinoma of the mamma: suggestions for a new method of treatment, with illustrative cases. Lancet 2 104–107. (10.1016/S0140-6736(01)72307-0) PMC5518378 29584099

[bib22] Beaver JA Park BH 2012 The BOLERO-2 trial: the addition of everolimus to exemestane in the treatment of postmenopausal hormone receptor-positive advanced breast cancer. Future Oncology 8 651–657. (10.2217/fon.12.49) 22764762 PMC3466807

[bib23] Beck JT Hortobagyi GN Campone M Lebrun F Deleu I Rugo HS Pistilli B Masuda N Hart L Melichar B 2014 Everolimus plus exemestane as first-line therapy in HR(+), HER2(−) advanced breast cancer in BOLERO-2. Breast Cancer Research and Treatment 143 459–467. (10.1007/s10549-013-2814-5) 24362951 PMC3907668

[bib24] Bentrem DJ Dardes RC Liu H Macgregor-Schafer J Zapf JW Jordan VC 2001 Molecular mechanism of action at estrogen receptor alpha of a new clinically revevant antiestrogen (GW7604) related to tamoxifen. Endocrinology 142 838–846. (10.1210/endo.142.2.7932) 11159857

[bib25] Berstein LM Wang JP Zheng H Yue W Conaway M Santen RJ 2004 Long-term exposure to tamoxifen induces hypersensitivity to estradiol. Clinical Cancer Research 10 1530–1534. (10.1158/1078-0432.CCR-0433-03) 14977857

[bib26] Bisson WH Abagyan R Cavasotto CN 2008 Molecular basis of agonicity and antagonicity in the androgen receptor studied by molecular dynamics simulations. Journal of Molecular Graphics 27 452–458. (10.1016/j.jmgm.2008.08.001) 18805032

[bib27] Bohl CE Gao W Miller DD Bell CE Dalton JT 2005a Structural basis for antagonism and resistance of bicalutamide in prostate cancer. PNAS 102 6201–6206. (10.1073/pnas.0500381102) 15833816 PMC1087923

[bib28] Bohl CE Miller DD Chen J Bell CE Dalton JT 2005b Structural basis for accommodation of nonsteroidal ligands in the androgen receptor. Journal of Biological Chemistry 280 37747–37754. (10.1074/jbc.M507464200) 16129672 PMC2072880

[bib29] Bolla M van Poppel H Collette L van Cangh P Vekemans K Da Pozzo L de Reijke TM Verbaeys A Bosset JF van Velthoven R 2005 Postoperative radiotherapy after radical prostatectomy: a randomised controlled trial (EORTC trial 22911). Lancet 366 572–578. (10.1016/S0140-6736(05)67101-2) 16099293

[bib30] Bosland MC 2016 Is there a future for chemoprevention of prostate cancer? Cancer Prevention Research 9 642–647. (10.1158/1940-6207.CAPR-16-0088) 27099271 PMC4970940

[bib31] Bostwick DG Grignon DJ Hammond ME Amin MB Cohen M Crawford D Gospadarowicz M Kaplan RS Miller DS Montironi R 2000 Prognostic factors in prostate cancer. College of American Pathologists Consensus Statement 1999. Archives of Pathology and Laboratory Medicine 124 995–1000. (10.1043/0003-9985(2000)124<0995:PFIPC>2.0.CO;2) 10888774

[bib32] Boyd S 1900 On oophorectomy in cancer of the breast. British Medical Journal 1900 1161–1167.

[bib33] Breast International Group (BIG) 1-98 Collaborative Group, Thurlimann B Keshaviah A Coates AS Mouridsen H Mauriac L Forbes JF Paridaens R Castiglione-Gertsch M Gelber RD 2005 A comparison of letrozole and tamoxifen in postmenopausal women with early breast cancer. New England Journal of Medicine 353 2747–2757. (10.1056/NEJMoa052258) 16382061

[bib34] Brodie AM Schwarzel WC Shaikh AA Brodie HJ 1977 The effect of an aromatase inhibitor, 4-hydroxy-4-androstene-3,17-dione, on estrogen-dependent processes in reproduction and breast cancer. Endocrinology 100 1684–1695. (10.1210/endo-100-6-1684) 404132

[bib35] Brodie AM Marsh D Brodie HJ 1979 Aromatase inhibitors – IV. Regression of hormone-dependent, mammary tumors in the rat with 4-acetoxy-4-androstene-3,17-dione. Journal of Steroid Biochemistry 10 423–429. (10.1016/0022-4731(79)90330-3) 109701

[bib36] Bruchovsky N Wilson JD 1968 The intranuclear binding of testosterone and 5-alpha-androstan-17-beta-ol-3-one by rat prostate. Journal of Biological Chemistry 243 5953–5960. 5696629

[bib37] Bruchovsky N Klotz LH Sadar M Crook JM Hoffart D Godwin L Warkentin M Gleave ME Goldenberg SL 2000 Intermittent androgen suppression for prostate cancer: Canadian Prospective Trial and related observations. Molecular Urology 4 191–199. 11062374

[bib38] Bubley GJ Balk SP 2017 Association between androgen receptor splice variants and prostate cancer resistance to abiraterone and enzalutamide. Journal of Clinical Oncology 35 2103–2105. (10.1200/JCO.2017.72.8808). 28414609

[bib39] Buchanan G Greenberg NM Scher HI Harris JM Marshall VR Tilley WD 2001 Collocation of androgen receptor gene mutations in prostate cancer. Clinical Cancer Research 7 1273–1281. 11350894

[bib40] Bulun SE Sharda G Rink J Sharma S Simpson ER 1996 Distribution of aromatase P450 transcripts and adipose fibroblasts in the human breast. Journal of Clinical Endocrinology and Metabolism 81 1273–1277. (10.1210/jcem.81.3.8772611) 8772611

[bib41] Busch S Sims AH Stal O Ferno M Landberg G 2015 Loss of TGFbeta receptor type 2 expression impairs estrogen response and confers tamoxifen resistance. Cancer Research 75 1457–1469. (10.1158/0008-5472.CAN-14-1583) 25833830

[bib42] Butt AJ McNeil CM Musgrove EA Sutherland RL 2005 Downstream targets of growth factor and oestrogen signalling and endocrine resistance: the potential roles of c-Myc, cyclin D1 and cyclin E. Endocrine-Related Cancer 12 (Supplement 1) S47–S59. (10.1677/erc.1.00993) 16113099

[bib43] Cai C Chen S Ng P Bubley GJ Nelson PS Mostaghel EA Marck B Matsumoto AM Simon NI Wang H 2011 Intratumoral *de novo* steroid synthesis activates androgen receptor in castration-resistant prostate cancer and is upregulated by treatment with CYP17A1 inhibitors. Cancer Research 71 6503–6513. (10.1158/0008-5472.CAN-11-0532) 21868758 PMC3209585

[bib44] Carey LA Perou CM 2015 Palbociclib – taking breast-cancer cells out of gear. New England Journal of Medicine 373 273–274. (10.1056/NEJMe1506680) 26176385

[bib45] Catalano S Giordano C Panza S Chemi F Bonofiglio D Lanzino M Rizza P Romeo F Fuqua SA Maggiolini M 2014 Tamoxifen through GPER upregulates aromatase expression: a novel mechanism sustaining tamoxifen-resistant breast cancer cell growth. Breast Cancer Research and Treatment 146 273–285. (10.1007/s10549-014-3017-4) 24928526

[bib46] Chang KH Ercole CE Sharifi N 2014 Androgen metabolism in prostate cancer: from molecular mechanisms to clinical consequences. British Journal of Cancer 111 1249–1254. (10.1038/bjc.2014.268) 24867689 PMC4183835

[bib47] Chen T Wang LH Farrar WL 2000 Interleukin 6 activates androgen receptor-mediated gene expression through a signal transducer and activator of transcription 3-dependent pathway in LNCaP prostate cancer cells. Cancer Research 60 2132–2135. 10786674

[bib48] Chen EJ Sowalsky AG Gao S Cai C Voznesensky O Schaefer R Loda M True LD Ye H Troncoso P 2015 Abiraterone treatment in castration-resistant prostate cancer selects for progesterone responsive mutant androgen receptors. Clinical Cancer Research 21 1273–1280. (10.1158/1078-0432.CCR-14-1220) 25320358 PMC4359958

[bib49] Chodak G Sharifi R Kasimis B Block NL Macramalla E Kennealey GT 1995 Single-agent therapy with bicalutamide: a comparison with medical or surgical castration in the treatment of advanced prostate carcinoma. Urology 46 849–855. (10.1016/S0090-4295(99)80356-2) 7502428

[bib50] Chu IM Hengst L Slingerland JM 2008 The Cdk inhibitor p27 in human cancer: prognostic potential and relevance to anticancer therapy. Nature Reviews Cancer 8 253–267. (10.1038/nrc2347) 18354415

[bib51] Chuu CP Kokontis JM Hiipakka RA Fukuchi J Lin HP Lin CY Huo C Su LC 2011a Androgens as therapy for androgen receptor-positive castration-resistant prostate cancer. Journal of Biomedical Science 18 63 (10.1186/1423-0127-18-63) 21859492 PMC3170584

[bib52] Chuu CP Kokontis JM Hiipakka RA Fukuchi J Lin HP Lin CY Huo C Su LC Liao S 2011b Androgen suppresses proliferation of castration-resistant LNCaP 104-R2 prostate cancer cells through androgen receptor, Skp2, and c-Myc. Cancer Science 102 2022–2028. (10.1111/j.1349-7006.2011.02043.x) 21781227 PMC3200457

[bib53] Cocconi G 1994 First generation aromatase inhibitors – aminoglutethimide and testololactone. Breast Cancer Research and Treatment 30 57–80. (10.1007/BF00682741) 7949205

[bib54] Colleoni M Luo W Karlsson P Chirgwin JH Aebi SP Jerusalem GHM Neven P Hitre E Graas M-P Simoncini E 2017 SOLE (Study of Letrozole Extension): a phase III randomized clinical trial of continuous vs intermittent letrozole in postmenopausal women who have received 4–6 years of adjuvant endocrine therapy for lymph node-positive, early breast cancer (BC). Journal of Clinical Oncology 35 503–503. (10.1200/JCO.2017.35.6_suppl.503)

[bib55] Coombes RC Goss P Dowsett M Gazet JC Brodie A 1984 4-Hydroxyandrostenedione in treatment of postmenopausal patients with advanced breast cancer. Lancet 2 1237–1239. (10.1016/S0140-6736(84)92795-8) 6150277

[bib56] Crawford ED Eisenberger MA Mcleod DG Spaulding JT Benson R Dorr FA Blumenstein BA Davis MA Goodman PJ 1989 A controlled trial of leuprolide with and without flutamide in prostatic-carcinoma. New England Journal of Medicine 321 419–424. (10.1056/NEJM198908173210702) 2503724

[bib57] Culig Z Hobisch A Cronauer MV Radmayr C Trapman J Hittmair A Bartsch G Klocker H 1994 Androgen receptor activation in prostatic tumor cell lines by insulin-like growth factor-I, keratinocyte growth factor, and epidermal growth factor. Cancer Research 54 5474–5478. 7522959

[bib58] Culig Z Bartsch G Hobisch A 2002 Interleukin-6 regulates androgen receptor activity and prostate cancer cell growth. Molecular and Cellular Endocrinology 197 231–238. (10.1016/S0303-7207(02)00263-0) 12431817

[bib59] Cummings SR Ensrud K Delmas PD LaCroix AZ Vukicevic S Reid DM Goldstein S Sriram U Lee A Thompson J 2010 Lasofoxifene in postmenopausal women with osteoporosis. New England Journal of Medicine 362 686–696. (10.1056/NEJMoa0808692) 20181970

[bib60] Cuzick J 2015 Statistical controversies in clinical research: long-term follow-up of clinical trials in cancer. Annals of Oncology 26 2363–2366. (10.1093/annonc/mdv392) 26433395 PMC4658544

[bib61] Cuzick J Forbes JF Sestak I Cawthorn S Hamed H Holli K Howell A & International Breast Cancer Intervention Study II 2007 Long-term results of tamoxifen prophylaxis for breast cancer – 96-month follow-up of the randomized IBIS-I trial. Journal of the National Cancer Institute 99 272–282. (10.1093/jnci/djk049) 17312304

[bib62] Cuzick J Sestak I Baum M Buzdar A Howell A Dowsett M Forbes JF Investigators AL 2010 Effect of anastrozole and tamoxifen as adjuvant treatment for early-stage breast cancer: 10-year analysis of the ATAC trial. Lancet Oncology 11 1135–1141. (10.1016/S1470-2045(10)70257-6) 21087898

[bib63] Cuzick J Wickerham L Powles T 2016 Differing perspectives on breast cancer chemoprevention. JAMA Oncology 2 276–277. (10.1001/jamaoncol.2015.4406) 26869000

[bib64] Davies C Godwin J Gray R Clarke M Darby S McGale P Wang YC Peto R Godwin J Pan HC 2011 Relevance of breast cancer hormone receptors and other factors to the efficacy of adjuvant tamoxifen: patient-level meta-analysis of randomised trials. Lancet 378 771–784. (10.1016/S0140-6736(11)60993-8) 21802721 PMC3163848

[bib65] Davies C Pan HC Godwin J Gray R Arriagada R Raina V Abraham M Alencar VHM Badran A Bonfill X 2013 Long-term effects of continuing adjuvant tamoxifen to 10 years versus stopping at 5 years after diagnosis of oestrogen receptor-positive breast cancer: ATLAS, a randomised trial. Lancet 381 805–816. (10.1016/S0140-6736(12)61963-1) 23219286 PMC3596060

[bib66] De Bono JS Oudard S Ozguroglu M Hansen S Machiels JP Kocak I Gravis G Bodrogi I Mackenzie MJ Shen L 2010 Prednisone plus cabazitaxel or mitoxantrone for metastatic castration-resistant prostate cancer progressing after docetaxel treatment: a randomised open-label trial. Lancet 376 1147–1154. (10.1016/S0140-6736(10)61389-X) 20888992

[bib67] De Bono JS Logothetis CJ Molina A Fizazi K North S Chu L Chi KN Jones RJ Goodman OB Jr Saad F 2011 Abiraterone and increased survival in metastatic prostate cancer. New England Journal of Medicine 364 1995–2005. (10.1056/NEJMoa1014618) 21612468 PMC3471149

[bib68] deGraffenried LA Friedrichs WE Russell DH Donzis EJ Middleton AK Silva JM Roth RA Hidalgo M 2004 Inhibition of mTOR activity restores tamoxifen response in breast cancer cells with aberrant Akt Activity. Clinical Cancer Research 10 8059–8067. (10.1158/1078-0432.CCR-04-0035) 15585641

[bib69] Dehm SM Schmidt LJ Heemers HV Vessella RL Tindall DJ 2008 Splicing of a novel androgen receptor exon generates a constitutively active androgen receptor that mediates prostate cancer therapy resistance. Cancer Research 68 5469–5477. (10.1158/0008-5472.CAN-08-0594) 18593950 PMC2663383

[bib70] Dosik M Kaufman R 2004 Raloxifene rebound regression. Cancer Investigation 22 718–722. (10.1081/CNV-200032978) 15581053

[bib71] Dowsett M Goss PE Powles TJ Hutchinson G Brodie AM Jeffcoate SL Coombes RC 1987 Use of the aromatase inhibitor 4-hydroxyandrostenedione in postmenopausal breast cancer: optimization of therapeutic dose and route. Cancer Research 47 1957–1961. 3815384

[bib72] Dowsett M Cuzick J Ingle J Coates A Forbes J Bliss J Buyse M Baum M Buzdar A Colleoni M 2010 Meta-analysis of breast cancer outcomes in adjuvant trials of aromatase inhibitors versus tamoxifen. Journal of Clinical Oncology 28 509–518. (10.1200/JCO.2009.23.1274) 19949017

[bib73] Duke CB Jones A Bohl CE Dalton JT Miller DD 2011 Unexpected binding orientation of bulky-B-ring anti-androgens and implications for future drug targets. Journal of Medicinal Chemistry 54 3973–3976. (10.1021/jm2000097) 21506597

[bib74] Early Breast Cancer Trialists’ Collaborative Group, Dowsett M Forbes JF Bradley R Ingle J Aihara T Bliss J Boccardo F Coates A Coombes RC 2015 Aromatase inhibitors versus tamoxifen in early breast cancer: patient-level meta-analysis of the randomised trials. Lancet 386 1341–1352. (10.1016/S0140-6736(15)61074-1) 26211827

[bib75] EBCTCG 1998 Tamoxifen for early breast cancer: an overview of the randomised trials. Early Breast Cancer Trialists’ Collaborative Group. Lancet 351 1451–1467. 9605801

[bib76] Edlind MP Hsieh AC 2014 PI3K-AKT-mTOR signaling in prostate cancer progression and androgen deprivation therapy resistance. Asian Journal of Andrology 16 378–386. (10.4103/1008-682X.122876) 24759575 PMC4023363

[bib77] Ellis MJ Gao F Dehdashti F Jeffe DB Marcom PK Carey LA Dickler MN Silverman P Fleming GF Kommareddy A 2009 Lower-dose vs high-dose oral estradiol therapy of hormone receptor-positive, aromatase inhibitor-resistant advanced breast cancer: a phase 2 randomized study. JAMA 302 774–780. (10.1001/jama.2009.1204) 19690310 PMC3460383

[bib78] Fan P McDaniel RE Kim HR Clagett D Haddad B Jordan VC 2012 Modulating therapeutic effects of the c-Src inhibitor via oestrogen receptor and human epidermal growth factor receptor 2 in breast cancer cell lines. European Journal of Cancer 48 3488–3498. (10.1016/j.ejca.2012.04.020) 22658320 PMC3518839

[bib79] Fan P Agboke FA Cunliffe HE Ramos P Jordan VC 2014a A molecular model for the mechanism of acquired tamoxifen resistance in breast cancer. European Journal of Cancer 50 2866–2876. (10.1016/j.ejca.2014.08.011) 25204804 PMC4194144

[bib80] Fan P Agboke FA McDaniel RE Sweeney EE Zou X Creswell K Jordan VC 2014b Inhibition of c-Src blocks oestrogen-induced apoptosis and restores oestrogen-stimulated growth in long-term oestrogen-deprived breast cancer cells. European Journal of Cancer 50 457–468. (10.1016/j.ejca.2013.10.001) 24183378 PMC3947251

[bib81] Fan P Cunliffe HE Griffith OL Agboke FA Ramos P Gray JW Jordan VC 2014c Identification of gene regulation patterns underlying both oestrogen- and tamoxifen-stimulated cell growth through global gene expression profiling in breast cancer cells. European Journal of Cancer 50 2877–2886. (10.1016/j.ejca.2014.08.010) 25212499 PMC4210771

[bib82] Fan P Cunliffe HE Maximov PY Agboke FA McDaniel RE Zou X Ramos P Russell ML Jordan VC 2015 Integration of downstream signals of insulin-like growth factor-1 receptor by endoplasmic reticulum stress for estrogen-induced growth or apoptosis in breast cancer cells. Molecular Cancer Research 13 1367–1376. (10.1158/1541-7786.MCR-14-0494) 26116171 PMC4763612

[bib83] Fanning SW Mayne CG Dharmarajan V Carlson KE Martin TA Novick SJ Toy W Green B Panchamukhi S Katzenellenbogen BS 2016 Estrogen receptor alpha somatic mutations Y537S and D538G confer breast cancer endocrine resistance by stabilizing the activating function-2 binding conformation. Elife 5 e12792 (10.7554/eLife.12792) 26836308 PMC4821807

[bib84] Fasolo A Sessa C 2008 mTOR inhibitors in the treatment of cancer. Expert Opinion on Investigational Drugs 17 1717–1734. (10.1517/13543784.17.11.1717) 18922108

[bib85] FDA 2011 FDA drug safety communication: 5-alpha reductase inhibitors (5-ARIs) may increase the risk of a more serious form of prostate cancer. Rockville, MD, USA: FDA (available at: https://www.fda.gov/Drugs/DrugSafety/ucm258314.htm)

[bib86] Feldman BJ Feldman D 2001 The development of androgen-independent prostate cancer. Nature Reviews Cancer 1 34–45. (10.1038/35094009) 11900250

[bib87] Finn RS Crown JP Lang I Boer K Bondarenko IM Kulyk SO Ettl J Patel R Pinter T Schmidt M 2015 The cyclin-dependent kinase 4/6 inhibitor palbociclib in combination with letrozole versus letrozole alone as first-line treatment of oestrogen receptor-positive, HER2-negative, advanced breast cancer (PALOMA-1/TRIO-18): a randomised phase 2 study. Lancet Oncology 16 25–35. (10.1016/S1470-2045(14)71159-3) 25524798

[bib88] Fisher B Costantino JP Wickerham DL Cecchini RS Cronin WM Robidoux A Bevers TB Kavanah MT Atkins JN Margolese RG 2005 Tamoxifen for the prevention of breast cancer: current status of the National Surgical Adjuvant Breast and Bowel Project P-1 study. Journal of the National Cancer Institute 97 1652–1662. (10.1093/jnci/dji372) 16288118

[bib89] Fleshner N Keane TE Lawton CA Mulders PF Payne H Taneja SS Morris T 2008 Adjuvant androgen deprivation therapy augments cure and long-term cancer control in men with poor prognosis, nonmetastatic prostate cancer. Prostate Cancer and Prostatic Diseases 11 46–52. (10.1038/sj.pcan.4500982) 17607304

[bib90] Fradet A Bouchet M Delliaux C Gervais M Kan C Benetollo C Pantano F Vargas G Bouazza L Croset M 2016 Estrogen related receptor alpha in castration-resistant prostate cancer cells promotes tumor progression in bone. Oncotarget 7 77071–77086. 27776343 10.18632/oncotarget.12787 PMC5363569

[bib91] Fujimoto N Mizokami A Harada S Matsumoto T 2001 Different expression of androgen receptor coactivators in human prostate. Urology 58 289–294. (10.1016/S0090-4295(01)01117-7) 11489729

[bib92] Furr BJ Jordan VC 1984 The pharmacology and clinical uses of tamoxifen. Pharmacology and Therapeutics 25 127–205. (10.1016/0163-7258(84)90043-3) 6438654

[bib93] Furr BJ Valcaccia B Curry B Woodburn JR Chesterson G Tucker H 1987 ICI 176,334: a novel non-steroidal, peripherally selective antiandrogen. Journal of Endocrinology 113 R7–R9. (10.1677/joe.0.113R007) 3625091

[bib94] Gleave M Tolcher A Miyake H Nelson C Brown B Beraldi E Goldie J 1999 Progression to androgen independence is delayed by adjuvant treatment with antisense Bcl-2 oligodeoxynucleotides after castration in the LNCaP prostate tumor model. Clinical Cancer Research 5 2891–2898. 10537358

[bib95] Goss PE Powles TJ Dowsett M Hutchison G Brodie AM Gazet JC Coombes RC 1986 Treatment of advanced postmenopausal breast cancer with an aromatase inhibitor, 4-hydroxyandrostenedione: phase II report. Cancer Research 46 4823–4826. 2942241

[bib96] Goss PE Ingle JN Martino S Robert NJ Muss HB Piccart MJ Castiglione M Tu D Shepherd LE Pritchard KI 2003 A randomized trial of letrozole in postmenopausal women after five years of tamoxifen therapy for early-stage breast cancer. New England Journal of Medicine 349 1793–1802. (10.1056/NEJMoa032312) 14551341

[bib97] Goss PE Ingle JN Martino S Robert NJ Muss HB Piccart MJ Castiglione M Tu D Shepherd LE Pritchard KI 2005 Randomized trial of letrozole following tamoxifen as extended adjuvant therapy in receptor-positive breast cancer: updated findings from NCIC CTG MA.17. Journal of the National Cancer Institute 97 1262–1271. (10.1093/jnci/dji250) 16145047

[bib98] Goss PE Ingle JN Pritchard KI Robert NJ Muss H Gralow J Gelmon K Whelan T Strasser-Weippl K Rubin S 2016 Extending aromatase-inhibitor adjuvant therapy to 10 years. New England Journal of Medicine 375 209–219. (10.1056/NEJMoa1604700) 27264120 PMC5024713

[bib99] Gottardis MM Jordan VC 1988 Development of tamoxifen-stimulated growth of MCF-7 tumors in athymic mice after long-term antiestrogen administration. Cancer Research 48 5183–5187. 3409244

[bib100] Gottardis MM Jiang SY Jeng MH Jordan VC 1989a Inhibition of tamoxifen-stimulated growth of an MCF-7 tumor variant in athymic mice by novel steroidal antiestrogens. Cancer Research 49 4090–4093. 2743303

[bib101] Gottardis MM Wagner RJ Borden EC Jordan VC 1989b Differential ability of antiestrogens to stimulate breast cancer cell (MCF-7) growth *in vivo* and *in vitro*. Cancer Research 49 4765–4769. 2758410

[bib102] Gray RG Rea D Handley K Bowden SJ Perry P Earl HM Poole CJ Bates T Chetiyawardana S Dewar JA 2013 aTTom: long-term effects of continuing adjuvant tamoxifen to 10 years versus stopping at 5 years in 6,953 women with early breast cancer. Journal of Clinical Oncology 31 (15 Supplement) abstract 5. (10.1200/jco.2013.31.15_suppl.5)

[bib103] Gregory CW He B Johnson RT Ford OH Mohler JL French FS Wilson EM 2001a A mechanism for androgen receptor-mediated prostate cancer recurrence after androgen deprivation therapy. Cancer Research 61 4315–4319. 11389051

[bib104] Gregory CW Johnson RT Jr Mohler JL French FS Wilson EM 2001b Androgen receptor stabilization in recurrent prostate cancer is associated with hypersensitivity to low androgen. Cancer Research 61 2892–2898. 11306464

[bib105] Grindstad T Andersen S Al-Saad S Donnem T Kiselev Y Nordahl Melbo-Jorgensen C Skjefstad K Busund LT Bremnes RM Richardsen E 2015 High progesterone receptor expression in prostate cancer is associated with clinical failure. PLoS ONE 10 e0116691 (10.1371/journal.pone.0116691) 25723513 PMC4344236

[bib106] Groner AC Cato L de Tribolet-Hardy J Bernasocchi T Janouskova H Melchers D Houtman R Cato AC Tschopp P Gu L 2016 TRIM24 is an oncogenic transcriptional activator in prostate cancer. Cancer Cell 29 846–858. (10.1016/j.ccell.2016.04.012) 27238081 PMC5124371

[bib107] Gu W Dong N Wang P Shi C Yang J Wang J 2017 Tamoxifen resistance and metastasis of human breast cancer cells were mediated by the membrane-associated estrogen receptor ER-alpha36 signaling *in vitro*. Cell Biology and Toxicology 33 183–195. (10.1007/s10565-016-9365-6) 27837347

[bib108] Gucalp A Traina TA 2016 Targeting the androgen receptor in triple-negative breast cancer. Current Problems in Cancer 40 141–150. (10.1016/j.currproblcancer.2016.09.004) 27816190 PMC5580391

[bib109] Gupta S Li J Kemeny G Bitting RL Beaver J Somarelli JA Ware KE Gregory S Armstrong AJ 2017 Whole genomic copy number alterations in circulating tumor cells from men with abiraterone or enzalutamide-resistant metastatic castration-resistant prostate cancer. Clinical Cancer Research 23 1346–1357. (10.1158/1078-0432.CCR-16-1211) 27601596

[bib110] Haddow A 1970 David A. Karnofsky memorial lecture. Thoughts on chemical therapy. Cancer 26 737–754. (10.1002/1097-0142(197010)26:4<737::AID-CNCR2820260402>3.0.CO;2-T) 4918638

[bib111] Haddow A Watkinson JM Paterson E Koller PC 1944 Influence of synthetic oestrogens on advanced malignant disease. BMJ 2 393–398. (10.1136/bmj.2.4368.393) 20785660 PMC2286289

[bib112] Hara T Miyazaki J Araki H Yamaoka M Kanzaki N Kusaka M Miyamoto M 2003 Novel mutations of androgen receptor: a possible mechanism of bicalutamide withdrawal syndrome. Cancer Research 63 149–153. 12517791

[bib113] Harvey JM Clark GM Osborne CK Allred DC 1999 Estrogen receptor status by immunohistochemistry is superior to the ligand-binding assay for predicting response to adjuvant endocrine therapy in breast cancer. Journal of Clinical Oncology 17 1474–1481. (10.1200/JCO.1999.17.5.1474) 10334533

[bib114] Hearn JW AbuAli G Reichard CA Reddy CA Magi-Galluzzi C Chang KH Carlson R Rangel L Reagan K Davis BJ 2016 HSD3B1 and resistance to androgen-deprivation therapy in prostate cancer: a retrospective, multicohort study. Lancet Oncology 17 1435–1444. (10.1016/S1470-2045(16)30227-3) 27575027 PMC5135009

[bib115] Hoimes CJ Kelly WK 2010 Redefining hormone resistance in prostate cancer. Therapeutic Advances in Medical Oncology 2 107–123. (10.1177/1758834009356433) 20543967 PMC2883184

[bib116] Homma Y Kaneko M Kondo Y Kawabe K Kakizoe T 1997 Inhibition of rat prostate carcinogenesis by a 5alpha-reductase inhibitor, FK143. Journal of the National Cancer Institute 89 803–807. (10.1093/jnci/89.11.803) 9182979

[bib117] Howell A Dodwell DJ Anderson H Redford J 1992 Response after withdrawal of tamoxifen and progestogens in advanced breast cancer. Annals of Oncology 3 611–617. (10.1093/oxfordjournals.annonc.a058286) 1450042

[bib118] Howell A Robertson JF Quaresma Albano J Aschermannova A Mauriac L Kleeberg UR Vergote I Erikstein B Webster A Morris C 2002 Fulvestrant, formerly ICI 182,780, is as effective as anastrozole in postmenopausal women with advanced breast cancer progressing after prior endocrine treatment. Journal of Clinical Oncology 20 3396–3403. (10.1200/JCO.2002.10.057) 12177099

[bib119] Huggins C Hodges CV 1941 Studies on prostatic cancer - I The effect of castration, of estrogen and of androgen injection on serum phosphatases in metastatic carcinoma of the prostate. Cancer Research 1 293–297. 10.3322/canjclin.22.4.232 4625049

[bib120] Ignatov A Ignatov T Roessner A Costa SD Kalinski T 2010 Role of GPR30 in the mechanisms of tamoxifen resistance in breast cancer MCF-7 cells. Breast Cancer Research and Treatment 123 87–96. (10.1007/s10549-009-0624-6) 19911269

[bib121] Ingle JN Ahmann DL Green SJ Edmonson JH Bisel HF Kvols LK Nichols WC Creagan ET Hahn RG Rubin J 1981 Randomized clinical trial of diethylstilbestrol versus tamoxifen in postmenopausal women with advanced breast cancer. New England Journal of Medicine 304 16–21. (10.1056/NEJM198101013040104) 7001242

[bib122] Jarrard DF Kinoshita H Shi Y Sandefur C Hoff D Meisner LF Chang C Herman JG Isaacs WB Nassif N 1998 Methylation of the androgen receptor promoter CpG island is associated with loss of androgen receptor expression in prostate cancer cells. Cancer Research 58 5310–5314. 9850055

[bib123] Jensen EV Jacobson HI 1962 Basic guides to the mechanism of estrogen action. Recent Progress in Hormone Research 18 387–414.

[bib124] Jeselsohn R Yelensky R Buchwalter G Frampton G Meric-Bernstam F Gonzalez-Angulo AM Ferrer-Lozano J Perez-Fidalgo JA Cristofanilli M Gomez H 2014 Emergence of constitutively active estrogen receptor-alpha mutations in pretreated advanced estrogen receptor-positive breast cancer. Clinical Cancer Research 20 1757–1767. (10.1158/1078-0432.CCR-13-2332) 24398047 PMC3998833

[bib125] Jiang SY Wolf DM Yingling JM Chang C Jordan VC 1992 An estrogen-receptor positive Mcf-7 clone that is resistant to antiestrogens and estradiol. Molecular and Cellular Endocrinology 90 77–86. (10.1016/0303-7207(92)90104-E) 1301400

[bib126] Joly-Pharaboz MO Ruffion A Roch A Michel-Calemard L Andre J Chantepie J Nicolas B Panaye G 2000 Inhibition of growth and induction of apoptosis by androgens of a variant of LNCaP cell line. Journal of Steroid Biochemistry and Molecular Biology 73 237–249. (10.1016/S0960-0760(00)00076-5) 11070352

[bib127] Jordan VC 1976 Effect of tamoxifen (ICI 46,474) on initiation and growth of DMBA-induced rat mammary carcinomata. European Journal of Cancer 12 419–424. (10.1016/0014-2964(76)90030-X) 821733

[bib128] Jordan VC 1978 DMBA-induced rat mammary carcinoma system for the evaluation of tamoxifen treatment as a potential adjuvant therapy. Reviews on Endocrine-Related Cancer (October Supplement) 49–55.

[bib129] Jordan VC 1984 Biochemical pharmacology of antiestrogen action. Pharmacological Reviews 36 245–276. 6395141

[bib130] Jordan VC 1991 Prolonged adjuvant tamoxifen: a beginning not the end. Annals of Oncology 2 481–484. (10.1093/oxfordjournals.annonc.a057995) 1911454

[bib131] Jordan VC 2003 Tamoxifen: a most unlikely pioneering medicine. Nature Reviews Drug Discovery 2 205–213. (10.1038/nrd1031) 12612646

[bib132] Jordan VC 2006 Tamoxifen (ICI46,474) as a targeted therapy to treat and prevent breast cancer. British Journal of Pharmacology 147 (Supplement 1) S269–S276. (10.1038/sj.bjp.0706399) 16402113 PMC1760730

[bib133] Jordan VC 2008 The 38th David A Karnofsky lecture: the paradoxical actions of estrogen in breast cancer--survival or death?. Journal of Clinical Oncology 26 3073–3082. (10.1200/JCO.2008.17.5190) 18519949

[bib134] Jordan VC 2014a Linking estrogen-induced apoptosis with decreases in mortality following long-term adjuvant tamoxifen therapy. Journal of the National Cancer Institute 106 dju296 (10.1093/jnci/dju296) 25269699 PMC4271028

[bib135] Jordan VC 2014b Tamoxifen as the first targeted long-term adjuvant therapy for breast cancer. Endocrine-Related Cancer 21 R235–R246. (10.1530/ERC-14-0092) 24659478 PMC4029058

[bib136] Jordan VC 2015a The new biology of estrogen-induced apoptosis applied to treat and prevent breast cancer. Endocrine-Related Cancer 22 R1–R31. (10.1530/ERC-14-0448) 25339261 PMC4494663

[bib137] Jordan VC 2015b Proven value of translational research with appropriate animal models to advance breast cancer treatment and save lives: the tamoxifen tale. British Journal of Clinical Pharmacology 79 254–267. (10.1111/bcp.12440) 24912921 PMC4309631

[bib138] Jordan VC 2016 Differing perspectives on breast cancer chemoprevention. JAMA Oncology 2 276 (10.1001/jamaoncol.2015.3906) 26868999

[bib139] Jordan VC 2017a The 4Ps of breast cancer chemoprevention: putting proven principles into practice. Cancer Prevention Research 10 219–222. (10.1158/1940-6207.CAPR-17-0026) 28246081 PMC5779859

[bib140] Jordan VC 2017b Concerns about methodology of a trial investigating vaginal health during aromatase inhibitor therapy for breast cancer. JAMA Oncology 3 1141 (10.1001/jamaoncol.2017.2074) 28727870

[bib141] Jordan VC Koerner S 1975 Tamoxifen (ICI 46,474) and the human carcinoma 8S oestrogen receptor. European Journal of Cancer 11 205–206. (10.1016/0014-2964(75)90119-X) 165942

[bib142] Jordan VC Allen KE 1980 Evaluation of the antitumour activity of the non-steroidal antioestrogen monohydroxytamoxifen in the DMBA-induced rat mammary carcinoma model. European Journal of Cancer 16 239–251. (10.1016/0014-2964(80)90156-5) 6768559

[bib143] Jordan VC Dix CJ Allen KE 1979 The effectiveness of long term tamoxifen treatment in a laboratory model for adjuvant hormone therapy of breast cancer. In Adjuavnt Therapy of Cancer II, pp 19–26. Eds Salmon SE Jones SE New York, NY, USA: Grune and Strattin.

[bib144] Jordan VC Naylor KE Dix CJ Prestwich G 1980 Anti-oestrogen action in experimental breast cancer. Recent Results in Cancer Research 71 30–44. 6768113 10.1007/978-3-642-81406-8_8

[bib145] Jordan VC Fritz NF Langan-Fahey S Thompson M Tormey DC 1991 Alteration of endocrine parameters in premenopausal women with breast cancer during long-term adjuvant therapy with tamoxifen as the single agent. Journal of the National Cancer Institute 83 1488–1491. (10.1093/jnci/83.20.1488) 1920495

[bib146] Jordan VC Curpan R Maximov PY 2015 Estrogen receptor mutations found in breast cancer metastases integrated with the molecular pharmacology of selective ER modulators. Journal of the National Cancer Institute 107 djv075 (10.1093/jnci/djv075) 25838462 PMC6390275

[bib147] Jordan VC Fan P Abderrahman B Maximov PY Hawsawi YM Bhattacharya P Pokharel N 2016 Sex steroid induced apoptosis as a rational strategy to treat anti-hormone resistant breast and prostate cancer. Discovery Medicine 21 411–427. 27355337

[bib148] Kaplan CP Haas JS Perez-Stable EJ Des Jarlais G Gregorich SE 2005 Factors affecting breast cancer risk reduction practices among California physicians. Preventive Medicine 41 7–15. (10.1016/j.ypmed.2004.09.041) 15916987

[bib149] Kattan MW Wheeler TM Scardino PT 1999 Postoperative nomogram for disease recurrence after radical prostatectomy for prostate cancer. Journal of Clinical Oncology 17 1499–1507. (10.1200/JCO.1999.17.5.1499) 10334537

[bib150] Katzenellenbogen BS Kendra KL Norman MJ Berthois Y 1987 Proliferation, hormonal responsiveness, and estrogen receptor content of MCF-7 human breast cancer cells grown in the short-term and long-term absence of estrogens. Cancer Research 47 4355–4360. 3607768

[bib151] Kawata H Ishikura N Watanabe M Nishimoto A Tsunenari T Aoki Y 2010 Prolonged treatment with bicalutamide induces androgen receptor overexpression and androgen hypersensitivity. Prostate 70 745–754. (10.1002/pros.21107) 20058237

[bib152] Kennedy BJ 1965 Hormone therapy for advanced breast cancer. Cancer 18 1551–1557. (10.1002/1097-0142(196512)18:12<1551::AID-CNCR2820181206>3.0.CO;2-1) 5845796

[bib153] Kirby M Hirst C Crawford ED 2011 Characterising the castration-resistant prostate cancer population: a systematic review. International Journal of Clinical Practice 65 1180–1192. (10.1111/j.1742-1241.2011.02799.x) 21995694

[bib154] Klotz L Higano CS 2016 Intermittent androgen deprivation therapy-an important treatment option for prostate cancer. JAMA Oncology 2 1531–1532. (10.1001/jamaoncol.2016.3138) 27560985

[bib155] Kokontis J Takakura K Hay N Liao S 1994 Increased androgen receptor activity and altered c-myc expression in prostate cancer cells after long-term androgen deprivation. Cancer Research 54 1566–1573. 7511045

[bib156] Kote-Jarai Z Leongamornlert D Saunders E Tymrakiewicz M Castro E Mahmud N Guy M Edwards S O’Brien L Sawyer E 2011 BRCA2 is a moderate penetrance gene contributing to young-onset prostate cancer: implications for genetic testing in prostate cancer patients. British Journal of Cancer 105 1230–1234. (10.1038/bjc.2011.383) 21952622 PMC3208504

[bib157] Labrie F Dupont A Giguere M Borsanyi JP Belanger A Lacourciere Y Emond J Monfette G 1986 Advantages of the combination therapy in previously untreated and treated patients with advanced prostate cancer. Journal of Steroid Biochemistry 25 877–883. (10.1016/0022-4731(86)90319-5) 3100871

[bib158] Legare S Basik M 2016 Minireview: the link between ERalpha corepressors and histone deacetylases in tamoxifen resistance in breast cancer. Molecular Endocrinology 30 965–976. (10.1210/me.2016-1072) 27581354 PMC5414611

[bib159] Lemmo W 2016 Anti-estrogen withdrawal effect with raloxifene? A case report. Integrative Cancer Therapies 15 245–249. (10.1177/1534735416658954) 27411856 PMC5739193

[bib160] Leongamornlert D Mahmud N Tymrakiewicz M Saunders E Dadaev T Castro E Goh C Govindasami K Guy M O’Brien L 2012 Germline BRCA1 mutations increase prostate cancer risk. British Journal of Cancer 106 1697–1701. (10.1038/bjc.2012.146) 22516946 PMC3349179

[bib161] Lerner LJ Jordan VC 1990 Development of antiestrogens and their use in breast cancer: eighth Cain memorial award lecture. Cancer Research 50 4177–4189. 2194650

[bib162] Lewis JS Meeke K Osipo C Ross EA Kidawi N Li T Bell E Chandel NS Jordan VC 2005a Intrinsic mechanism of estradiol-induced apoptosis in breast cancer cells resistant to estrogen deprivation. Journal of the National Cancer Institute 97 1746–1759. (10.1093/jnci/dji400) 16333030

[bib163] Lewis JS Osipo C Meeke K Jordan VC 2005b Estrogen-induced apoptosis in a breast cancer model resistant to long-term estrogen withdrawal. Journal of Steroid Biochemistry and Molecular Biology 94 131–141. (10.1016/j.jsbmb.2004.12.032) 15862958

[bib164] Li J Yen C Liaw D Podsypanina K Bose S Wang SI Puc J Miliaresis C Rodgers L McCombie R 1997 PTEN, a putative protein tyrosine phosphatase gene mutated in human brain, breast, and prostate cancer. Science 275 1943–1947. (10.1126/science.275.5308.1943) 9072974

[bib165] Lin HK Yeh S Kang HY Chang C 2001 Akt suppresses androgen-induced apoptosis by phosphorylating and inhibiting androgen receptor. PNAS 98 7200–7205. (10.1073/pnas.121173298) 11404460 PMC34646

[bib166] Lipton A Santen RJ 1974 Proceedings: medical adrenalectomy using aminoglutethimide and dexamethasone in advanced breast cancer. Cancer 33 503–512. (10.1002/1097-0142(197402)33:2<503::AID-CNCR2820330227>3.0.CO;2-L) 4812767

[bib167] Locke JA Guns ES Lubik AA Adomat HH Hendy SC Wood CA Ettinger SL Gleave ME Nelson CC 2008 Androgen levels increase by intratumoral de novo steroidogenesis during progression of castration-resistant prostate cancer. Cancer Research 68 6407–6415. (10.1158/0008-5472.CAN-07-5997) 18676866

[bib168] Lu-Yao GL Albertsen PC Moore DF Shih W Lin Y DiPaola RS Barry MJ Zietman A O’Leary M Walker-Corkery E 2009 Outcomes of localized prostate cancer following conservative management. JAMA 302 1202–1209. (10.1001/jama.2009.1348) 19755699 PMC2822438

[bib169] Mainwaring WI 1969 A soluble androgen receptor in the cytoplasm of rat prostate. Journal of Endocrinology 45 531–541. (10.1677/joe.0.0450531) 5366114

[bib170] Marcelli M Ittmann M Mariani S Sutherland R Nigam R Murthy L Zhao Y DiConcini D Puxeddu E Esen A 2000 Androgen receptor mutations in prostate cancer. Cancer Research 60 944–949. 10706109

[bib171] Marques RB Dits NF Erkens-Schulze S van Weerden WM Jenster G 2010 Bypass mechanisms of the androgen receptor pathway in therapy-resistant prostate cancer cell models. PLoS ONE 5 e13500 (10.1371/journal.pone.0013500) 20976069 PMC2957443

[bib172] Masamura S Santner SJ Heitjan DF Santen RJ 1995 Estrogen deprivation causes estradiol hypersensitivity in human breast cancer cells. Journal of Clinical Endocrinology and Metabolism 80 2918–2925. (10.1210/jcem.80.10.7559875) 7559875

[bib173] Mathew P 2008 Prolonged control of progressive castration-resistant metastatic prostate cancer with testosterone replacement therapy: the case for a prospective trial. Annals of Oncology 19 395–396. (10.1093/annonc/mdm568) 18156142

[bib174] Maximov PY Lee TM Jordan VC 2013 The discovery and development of selective estrogen receptor modulators (SERMs) for clinical practice. Current Clinical Pharmacology 8 135–155. (10.2174/1574884711308020006) 23062036 PMC3624793

[bib175] McGuire WL Carbone PP Vollmer EP, United States National Cancer Institute & Breast Cancer Treatment Committee 1975 Estrogen Receptors in Human Breast Cancer. New York: Raven Press.

[bib176] Melisko ME Goldman ME Hwang J De Luca A Fang S Esserman LJ Chien AJ Park JW Rugo HS 2017 Vaginal testosterone cream vs estradiol vaginal ring for vaginal dryness or decreased libido in women receiving aromatase inhibitors for early-stage breast cancer: a randomized clinical trial. JAMA Oncology 3 313–319. (10.1001/jamaoncol.2016.3904) 27832260

[bib177] Miller TW 2013 Endocrine resistance: what do we know? American Society of Clinical Oncology Educational Book. (10.1200/EdBook_AM.2013.33.e37) 23714450

[bib178] Mishra S Tai Q Gu X Schmitz J Poullard A Fajardo RJ Mahalingam D Chen X Zhu X Sun LZ 2015 Estrogen and estrogen receptor alpha promotes malignancy and osteoblastic tumorigenesis in prostate cancer. Oncotarget 6 44388–44402. (10.18632/oncotarget.6317) 26575018 PMC4792564

[bib179] Mo Z Liu M Yang F Luo H Li Z Tu G Yang G 2013 GPR30 as an initiator of tamoxifen resistance in hormone-dependent breast cancer. Breast Cancer Research 15 R114 (10.1186/bcr3581) 24289103 PMC3978564

[bib180] Mulholland DJ Tran LM Li Y Cai H Morim A Wang S Plaisier S Garraway IP Huang J Graeber TG 2011 Cell autonomous role of PTEN in regulating castration-resistant prostate cancer growth. Cancer Cell 19 792–804. (10.1016/j.ccr.2011.05.006) 21620777 PMC3157296

[bib181] Navarro D Luzardo OP Fernandez L Chesa N Diaz-Chico BN 2002 Transition to androgen-independence in prostate cancer. Journal of Steroid Biochemistry and Molecular Biology 81 191–201. (10.1016/S0960-0760(02)00064-X) 12163131

[bib182] Neri R Florance K Koziol P Van Cleave S 1972 A biological profile of a nonsteroidal antiandrogen, SCH 13521 (4'-nitro-3'trifluoromethylisobutyranilide). Endocrinology 91 427–437. (10.1210/endo-91-2-427) 4264731

[bib183] Nettles KW Bruning JB Gil G Nowak J Sharma SK Hahm JB Kulp K Hochberg RB Zhou H Katzenellenbogen JA 2008 NFkappaB selectivity of estrogen receptor ligands revealed by comparative crystallographic analyses. Nature Chemical Biology 4 241–247. (10.1038/nchembio.76) 18344977 PMC2659626

[bib184] Ni L Yang CS Gioeli D Frierson H Toft DO Paschal BM 2010 FKBP51 promotes assembly of the Hsp90 chaperone complex and regulates androgen receptor signaling in prostate cancer cells. Molecular and Cellular Biology 30 1243–1253. (10.1128/MCB.01891-08) 20048054 PMC2820886

[bib185] Nishiyama T Hashimoto Y Takahashi K 2004 The influence of androgen deprivation therapy on dihydrotestosterone levels in the prostatic tissue of patients with prostate cancer. Clinical Cancer Research 10 7121–7126. (10.1158/1078-0432.CCR-04-0913) 15534082

[bib186] O’Leary B Finn RS Turner NC 2016 Treating cancer with selective CDK4/6 inhibitors. Nature Reviews Clinical Oncology 13 417–430. (10.1038/nrclinonc.2016.26) 27030077

[bib187] Omlin A Jones RJ van der Noll R Satoh T Niwakawa M Smith SA Graham J Ong M Finkelman RD Schellens JH 2015 AZD3514, an oral selective androgen receptor down-regulator in patients with castration-resistant prostate cancer – results of two parallel first-in-human phase I studies. Investigational New Drugs 33 679–690. (10.1007/s10637-015-0235-5) 25920479

[bib188] Omrcen T Hrepic D Boraska Jelavic T Vrdoljak E 2015 Combination of adjuvant radiotherapy and androgen deprivation therapy after radical prostatectomy in high risk prostate cancer patients – results from retrospective analysis. Journal of BUON 20 1061–1067. 26416057

[bib189] Osborne CK Coronado EB Robinson JP 1987 Human breast cancer in the athymic nude mouse: cytostatic effects of long-term antiestrogen therapy. European Journal of Cancer and Clinical Oncology 23 1189–1196. (10.1016/0277-5379(87)90154-4) 3653212

[bib190] Osborne CK Pippen J Jones SE Parker LM Ellis M Come S Gertler SZ May JT Burton G Dimery I 2002 Double-blind, randomized trial comparing the efficacy and tolerability of fulvestrant versus anastrozole in postmenopausal women with advanced breast cancer progressing on prior endocrine therapy: results of a North American trial. Journal of Clinical Oncology 20 3386–3395. (10.1200/JCO.2002.10.058) 12177098

[bib191] Osborne CK Bardou V Hopp TA Chamness GC Hilsenbeck SG Fuqua SA Wong J Allred DC Clark GM Schiff R 2003 Role of the estrogen receptor coactivator AIB1 (SRC-3) and HER-2/neu in tamoxifen resistance in breast cancer. Journal of the National Cancer Institute 95 353–361. (10.1093/jnci/95.5.353) 12618500

[bib192] Owens WL Gallagher TJ Kincheloe MJ Ruetten VL 2011 Implementation in a large health system of a program to identify women at high risk for breast cancer. Journal of Oncology Practice 7 85–88. (10.1200/JOP.2010.000107) 21731514 PMC3051867

[bib193] Pan H Gray R Davies C 2016 Predictors of recurrence during years 5-14 in 46,138 women with ER+ breast cancer allocated 5 years only of endocrine therapy (ET). Journal of Clinical Oncology 34 (15 Supplement) 505 (10.1200/JCO.2016.34.15_suppl.505)

[bib194] Pan H Gray R Braybrooke J Davies C Taylor C McGale P Peto R Pritchard KI Bergh J Dowsett M 2017 20-year risks of breast-cancer recurrence after stopping endocrine therapy at 5 years. New England Journal of Medicine 377 1836–1846. (10.1056/NEJMoa1701830) 29117498 PMC5734609

[bib195] Papadopoulou N Charalampopoulos I Alevizopoulos K Gravanis A Stournaras C 2008a Rho/ROCK/actin signaling regulates membrane androgen receptor induced apoptosis in prostate cancer cells. Experimental Cell Research 314 3162–3174. (10.1016/j.yexcr.2008.07.012) 18694745

[bib196] Papadopoulou N Charalampopoulos I Anagnostopoulou V Konstantinidis G Foller M Gravanis A Alevizopoulos K Lang F Stournaras C 2008b Membrane androgen receptor activation triggers down-regulation of PI-3K/Akt/NF-kappaB activity and induces apoptotic responses via Bad, FasL and caspase-3 in DU145 prostate cancer cells. Molecular Cancer 7 88 (10.1186/1476-4598-7-88) 19055752 PMC2629475

[bib197] Papakonstanti EA Kampa M Castanas E Stournaras C 2003 A rapid, nongenomic, signaling pathway regulates the actin reorganization induced by activation of membrane testosterone receptors. Molecular Endocrinology 17 870–881. (10.1210/me.2002-0253) 12554777

[bib198] Payne H Mason M 2011 Androgen deprivation therapy as adjuvant/neoadjuvant to radiotherapy for high-risk localised and locally advanced prostate cancer: recent developments. British Journal of Cancer 105 1628–1634. (10.1038/bjc.2011.385) 22009028 PMC3242586

[bib199] Peethambaram PP Ingle JN Suman VJ Hartmann LC Loprinzi CL 1999 Randomized trial of diethylstilbestrol vs. tamoxifen in postmenopausal women with metastatic breast cancer. An updated analysis. Breast Cancer Research and Treatment 54 117–122. (10.1023/A:1006185805079) 10424402

[bib200] Penson DF Armstrong AJ Concepcion R Agarwal N Olsson C Karsh L Dunshee C Wang F Wu K Krivoshik A 2016 Enzalutamide versus bicalutamide in castration-resistant prostate cancer: the STRIVE trial. Journal of Clinical Oncology 34 2098–2106. (10.1200/JCO.2015.64.9285) 26811535

[bib201] Perez-Tenorio G Berglund F Esguerra Merca A Nordenskjold B Rutqvist LE Skoog L Stal O 2006 Cytoplasmic p21WAF1/CIP1 correlates with Akt activation and poor response to tamoxifen in breast cancer. International Journal of Oncology 28 1031–1042. (10.3892/ijo.28.5.1031) 16596219

[bib202] Pether M Goldenberg SL Bhagirath K Gleave M 2003 Intermittent androgen suppression in prostate cancer: an update of the Vancouver experience. Canadian Journal of Urology 10 1809–1814. 12773232

[bib203] Phallen J Sausen M Adleff V Leal A Hruban C White J Anagnostou V Fiksel J Cristiano S Papp E 2017 Direct detection of early-stage cancers using circulating tumor DNA. Science Translational Medicine 9. 10.1126/scitranslmed.aan2415 PMC6714979 28814544

[bib204] Pink JJ Jiang SY Fritsch M Jordan VC 1995 An estrogen-independent MCF-7 breast cancer cell line which contains a novel 80-kilodalton estrogen receptor-related protein. Cancer Research 55 2583–2590. 7780972

[bib205] Powles TJ Ashley S Tidy A Smith IE Dowsett M 2007 Twenty-year follow-up of the Royal Marsden randomized, double-blinded tamoxifen breast cancer prevention trial. Journal of the National Cancer Institute 99 283–290. (10.1093/jnci/djk050) 17312305

[bib206] Prat A Perou CM 2011 Deconstructing the molecular portraits of breast cancer. Molecular Oncology 5 5–23. (10.1016/j.molonc.2010.11.003) 21147047 PMC5528267

[bib207] Pritchard KI Chia SK Simmons C McLeod D Paterson A Provencher L Rayson D 2017 Enhancing endocrine therapy combination strategies for the treatment of postmenopausal HR+/HER2- advanced breast cancer. Oncologist 22 12–24. (10.1634/theoncologist.2016-0185) 27864574 PMC5313264

[bib208] Rathkopf DE Morris MJ Danila DC Slovin SF Steinbrecher JE Arauz G Curley T Rix PJ Maneval EC Chen I 2012 A phase I study of the androgen signaling inhibitor ARN-509 in patients with metastatic castration-resistant prostate cancer (mCRPC). Journal of Clinical Oncology 30 (15 Supplement) 4548–4548. (10.1200/jco.2012.30.15_suppl.4548)

[bib209] Rathkopf DE Morris MJ Fox JJ Danila DC Slovin SF Hager JH Rix PJ Chow Maneval E Chen I Gonen M 2013 Phase I study of ARN-509, a novel antiandrogen, in the treatment of castration-resistant prostate cancer. Journal of Clinical Oncology 31 3525–3530. (10.1200/JCO.2013.50.1684) 24002508 PMC3782148

[bib210] Rau KM Kang HY Cha TL Miller SA Hung MC 2005 The mechanisms and managements of hormone-therapy resistance in breast and prostate cancers. Endocrine-Related Cancer 12 511–532. (10.1677/erc.1.01026) 16172190

[bib211] Ravdin PM Fritz NF Tormey DC Jordan VC 1988 Endocrine status of premenopausal node-positive breast cancer patients following adjuvant chemotherapy and long-term tamoxifen. Cancer Research 48 1026–1029. 3123050

[bib212] Raynaud JP Bonne C Bouton MM Lagace L Labrie F 1979 Action of a non-steroid anti-androgen, RU 23908, in peripheral and central tissues. Journal of Steroid Biochemistry 11 93–99. (10.1016/0022-4731(79)90281-4) 385986

[bib213] Reeder-Hayes K Muss HB 2017 Vaginal estrogens and aromatase inhibitors: how safe is safe enough? JAMA Oncology 3 305–306. (10.1001/jamaoncol.2016.3934) 27832252

[bib214] Riggins RB Bouton AH Liu MC Clarke R 2005 Antiestrogens, aromatase inhibitors, and apoptosis in breast cancer. Vitamins and Hormones 71 201–237. 16112269 10.1016/S0083-6729(05)71007-4

[bib215] Risbridger GP Davis ID Birrell SN Tilley WD 2010 Breast and prostate cancer: more similar than different. Nature Reviews Cancer 10 205–212. (10.1038/nrc2795) 20147902

[bib216] Robertson JFR Bondarenko IM Trishkina E Dvorkin M Panasci L Manikhas A Shparyk Y Cardona-Huerta S Cheung KL Philco-Salas MJ 2016 Fulvestrant 500 mg versus anastrozole 1 mg for hormone receptor-positive advanced breast cancer (FALCON): an international, randomised, double-blind, phase 3 trial. Lancet 388 2997–3005. (10.1016/S0140-6736(16)32389-3) 27908454

[bib217] Robinson DR Wu YM Vats P Su F Lonigro RJ Cao X Kalyana-Sundaram S Wang R Ning Y Hodges L 2013 Activating ESR1 mutations in hormone-resistant metastatic breast cancer. Nature Genetics 45 1446–1451. (10.1038/ng.2823) 24185510 PMC4009946

[bib218] Robinson D Van Allen EM Wu YM Schultz N Lonigro RJ Mosquera JM Montgomery B Taplin ME Pritchard CC Attard G 2015 Integrative clinical genomics of advanced prostate cancer. Cell 161 1215–1228. (10.1016/j.cell.2015.05.001) 26000489 PMC4484602

[bib219] Ropke A Erbersdobler A Hammerer P Palisaar J John K Stumm M Wieacker P 2004 Gain of androgen receptor gene copies in primary prostate cancer due to X chromosome polysomy. Prostate 59 59–68. (10.1002/pros.10356) 14991866

[bib220] Rosen EM Fan S Goldberg ID 2001 BRCA1 and prostate cancer. Cancer Investigation 19 396–412. (10.1081/CNV-100103134) 11405179

[bib221] Rossi L Pagani O 2017 Adjuvant endocrine therapy in breast cancer: evolving paradigms in premenopausal women. Current Treatment Options in Oncology 18 28 (10.1007/s11864-017-0473-1) 28439796

[bib222] Ruijter E van de Kaa C Miller G Ruiter D Debruyne F Schalken J 1999 Molecular genetics and epidemiology of prostate carcinoma. Endocrine Reviews 20 22–45. (10.1210/edrv.20.1.0356) 10047972

[bib223] Sahu B Laakso M Pihlajamaa P Ovaska K Sinielnikov I Hautaniemi S Janne OA 2013 FoxA1 specifies unique androgen and glucocorticoid receptor binding events in prostate cancer cells. Cancer Research 73 1570–1580. (10.1158/0008-5472.CAN-12-2350) 23269278

[bib224] Santen RJ Worgul TJ Samojlik E Interrante A Boucher AE Lipton A Harvey HA White DS Smart E Cox C 1981 A randomized trial comparing surgical adrenalectomy with aminoglutethimide plus hydrocortisone in women with advanced breast cancer. New England Journal of Medicine 305 545–551. (10.1056/NEJM198109033051003) 7019703

[bib225] Santen RJ Song RX Zhang Z Kumar R Jeng MH Masamura S Yue W Berstein L 2003 Adaptive hypersensitivity to estrogen: mechanism for superiority of aromatase inhibitors over selective estrogen receptor modulators for breast cancer treatment and prevention. Endocrine-Related Cancer 10 111–130. (10.1677/erc.0.0100111) 12790774

[bib226] Santen RJ Brodie H Simpson ER Siiteri PK Brodie A 2009a History of aromatase: saga of an important biological mediator and therapeutic target. Endocrine Reviews 30 343–375. (10.1210/er.2008-0016) 19389994

[bib227] Santen RJ Fan P Zhang Z Bao Y Song RX Yue W 2009b Estrogen signals via an extra-nuclear pathway involving IGF-1R and EGFR in tamoxifen-sensitive and -resistant breast cancer cells. Steroids 74 586–594. (10.1016/j.steroids.2008.11.020) 19138696

[bib228] Sato N Gleave ME Bruchovsky N Rennie PS Goldenberg SL Lange PH Sullivan LD 1996 Intermittent androgen suppression delays progression to androgen-independent regulation of prostate-specific antigen gene in the LNCaP prostate tumour model. Journal of Steroid Biochemistry and Molecular Biology 58 139–146. (10.1016/0960-0760(96)00018-0) 8809195

[bib229] Schally AV Kastin AJ Arimura A 1971 Hypothalamic follicle-stimulating hormone (FSH) and luteinizing hormone (LH)-regulating hormone: structure, physiology, and clinical studies. Fertility and Sterility 22 703–721. (10.1016/S0015-0282(16)38580-6) 4941683

[bib230] Schally AV Kastin AJ Arimura A 1971 Hypothalamic follicle-stimulating hormone (FSH) and luteinizing hormone (LH)-regulating hormone: structure, physiology, and clinical studies. Fertility and Sterility 22 703–721. (10.1016/S0015-0282(16)38580-6) 4941683

[bib231] Schally AV Comaru-Schally AM Plonowski A Nagy A Halmos G Rekasi Z 2000 Peptide analogs in the therapy of prostate cancer. Prostate 45 158–166. (10.1002/1097-0045(20001001)45:2<158::AID-PROS10>3.0.CO;2-K) 11027415

[bib232] Scher HI Beer TM Higano CS Anand A Taplin ME Efstathiou E Rathkopf D Shelkey J Yu EY Alumkal J 2010 Antitumour activity of MDV3100 in castration-resistant prostate cancer: a phase 1-2 study. Lancet 375 1437–1446. (10.1016/S0140-6736(10)60172-9) 20398925 PMC2948179

[bib233] Schiff R Massarweh SA Shou J Bharwani L Mohsin SK Osborne CK 2004 Cross-talk between estrogen receptor and growth factor pathways as a molecular target for overcoming endocrine resistance. Clinical Cancer Research 10 331S–336S. (10.1158/1078-0432.CCR-031212) 14734488

[bib234] Schrey MP Patel KV 1995 Prostaglandin E2 production and metabolism in human breast cancer cells and breast fibroblasts. Regulation by inflammatory mediators. British Journal of Cancer 72 1412–1419. (10.1038/bjc.1995.523) 8519653 PMC2034098

[bib235] Schwartz GK LoRusso PM Dickson MA Randolph SS Shaik MN Wilner KD Courtney R O’Dwyer PJ 2011 Phase I study of PD 0332991, a cyclin-dependent kinase inhibitor, administered in 3-week cycles (Schedule 2/1). British Journal of Cancer 104 1862–1868. (10.1038/bjc.2011.177) 21610706 PMC3111206

[bib236] Schweizer MT Antonarakis ES Wang H Ajiboye AS Spitz A Cao H Luo J Haffner MC Yegnasubramanian S Carducci MA 2015 Effect of bipolar androgen therapy for asymptomatic men with castration-resistant prostate cancer: results from a pilot clinical study. Science Translational Medicine 7 269ra2 (10.1126/scitranslmed.3010563) PMC4507510 25568070

[bib237] Seidenfeld J Samson DJ Hasselblad V Aronson N Albertsen PC Bennett CL Wilt TJ 2000 Single-therapy androgen suppression in men with advanced prostate cancer: a systematic review and meta-analysis. Annals of Internal Medicine 132 566–577. (10.7326/0003-4819-132-7-200004040-00009) 10744594

[bib238] Shipley WU Seiferheld W Lukka HR Major PP Heney NM Grignon DJ Sartor O Patel MP Bahary JP Zietman AL 2017 Radiation with or without antiandrogen therapy in recurrent prostate cancer. New England Journal of Medicine 376 417–428. (10.1056/NEJMoa1607529) 28146658 PMC5444881

[bib239] Shou J Massarweh S Osborne CK Wakeling AE Ali S Weiss H Schiff R 2004 Mechanisms of tamoxifen resistance: increased estrogen receptor-HER2/neu cross-talk in ER/HER2-positive breast cancer. Journal of the National Cancer Institute 96 926–935. (10.1093/jnci/djh166) 15199112

[bib240] Siegel RL Miller KD Jemal A 2015 Cancer statistics, 2015. CA: A Cancer Journal for Clinicians 65 5–29. (10.3322/caac.21254) 25559415

[bib241] Sledge GW Mamounas EP Hortobagyi GN Burstein HJ Goodwin PJ Wolff AC 2014 Past, present, and future challenges in breast cancer treatment. Journal of Clinical Oncology 32 1979–1986. (10.1200/JCO.2014.55.4139) 24888802 PMC4879690

[bib242] Smith DC Smith MR Sweeney C Elfiky AA Logothetis C Corn PG Vogelzang NJ Small EJ Harzstark AL Gordon MS 2013 Cabozantinib in patients with advanced prostate cancer: results of a phase II randomized discontinuation trial. Journal of Clinical Oncology 31 412–419. (10.1200/JCO.2012.45.0494) 23169517 PMC4110249

[bib243] Smith SG Side L Meisel SF Horne R Cuzick J Wardle J 2016 Clinician-reported barriers to implementing breast cancer chemoprevention in the UK: a qualitative investigation. Public Health Genomics 19 239–249. (10.1159/000447552) 27399355

[bib244] Smith SG Sestak I Howell A Forbes J Cuzick J 2017 Participant-reported symptoms and their effect on long-term adherence in the international breast cancer intervention study I (IBIS I). Journal of Clinical Oncology 10 2666–2673. (10.1200/JCO.2016.71.7439) PMC5549455 28661758

[bib245] Song RXD Mor G Naftolin F McPherson RA Song J Zhang ZG Yue W Wang JP Santen RJ 2001 Effect of long-term estrogen deprivation on apoptotic responses of breast cancer cells to 17 beta-estradiol. Journal of the National Cancer Institute 93 1714–1723. (10.1093/jnci/93.22.1714) 11717332

[bib246] Span PN Tjan-Heijnen VC Manders P Beex LV Sweep CG 2003 Cyclin-E is a strong predictor of endocrine therapy failure in human breast cancer. Oncogene 22 4898–4904. (10.1038/sj.onc.1206818) 12894232

[bib247] Stoll BA 1991 Overprolonged adjuvant tamoxifen therapy in breast cancer. Annals of Oncology 2 401–403. (10.1093/oxfordjournals.annonc.a057973) 1768625

[bib248] Suen CS Berrodin TJ Mastroeni R Cheskis BJ Lyttle CR Frail DE 1998 A transcriptional coactivator, steroid receptor coactivator-3, selectively augments steroid receptor transcriptional activity. Journal of Biological Chemistry 273 27645–27653. (10.1074/jbc.273.42.27645) 9765300

[bib249] Sun B Ding L Wu S Meng X Song S 2016 Combined treatment with everolimus and fulvestrant reversed anti-HER2 resistance in a patient with refractory advanced breast cancer: a case report. OncoTargets and Therapy 9 3997–4003. (10.2147/OTT.S104398) 27445490 PMC4936809

[bib250] Suzuki H Ueda T Ichikawa T Ito H 2003 Androgen receptor involvement in the progression of prostate cancer. Endocrine-Related Cancer 10 209–216. (10.1677/erc.0.0100209) 12790784

[bib251] Tan MH Li J Xu HE Melcher K Yong EL 2015 Androgen receptor: structure, role in prostate cancer and drug discovery. Acta Pharmacologica Sinica 36 3–23. (10.1038/aps.2014.18) 24909511 PMC4571323

[bib252] Taylor BS Schultz N Hieronymus H Gopalan A Xiao Y Carver BS Arora VK Kaushik P Cerami E Reva B 2010 Integrative genomic profiling of human prostate cancer. Cancer Cell 18 11–22. (10.1016/j.ccr.2010.05.026) 20579941 PMC3198787

[bib253] Theoret MR Ning YM Zhang JJ Justice R Keegan P Pazdur R 2011 The risks and benefits of 5alpha-reductase inhibitors for prostate-cancer prevention. New England Journal of Medicine 365 97–99. (10.1056/NEJMp1106783) 21675880

[bib254] Thorpe JF Jain S Marczylo TH Gescher AJ Steward WP Mellon JK 2007 A review of phase III clinical trials of prostate cancer chemoprevention. Annals of the Royal College of Surgeons of England 89 207–211. (10.1308/003588407X179125) 17394699 PMC1964727

[bib255] Titus MA Schell MJ Lih FB Tomer KB Mohler JL 2005 Testosterone and dihydrotestosterone tissue levels in recurrent prostate cancer. Clinical Cancer Research 11 4653–4657. (10.1158/1078-0432.CCR-05-0525) 16000557

[bib256] Toft D Gorski J 1966 A receptor molecule for estrogens: isolation from the rat uterus and preliminary characterization. PNAS 55 1574–1581. (10.1073/pnas.55.6.1574) 5227676 PMC224361

[bib257] Tolis G Ackman D Stellos A Mehta A Labrie F Fazekas ATA Comaruschally AM Schally AV 1982 Tumor-Growth Inhibition in Patients with Prostatic-Carcinoma Treated with Luteinizing-Hormone-Releasing Hormone Agonists. PNAS 79 1658–1662. (10.1073/pnas.79.5.1658) 6461861 PMC346035

[bib258] Tomiguchi M Yamamoto Y Yamamoto-Ibusuki M Goto-Yamaguchi L Fujiki Y Fujiwara S Sueta A Hayashi M Takeshita T Inao T 2016 Fibroblast growth factor receptor-1 protein expression is associated with prognosis in estrogen receptor-positive/human epidermal growth factor receptor-2-negative primary breast cancer. Cancer Science 107 491–498. (10.1111/cas.12897) 26801869 PMC4832856

[bib259] Toy W Shen Y Won H Green B Sakr RA Will M Li Z Gala K Fanning S King TA 2013 ESR1 ligand-binding domain mutations in hormone-resistant breast cancer. Nature Genetics 45 1439–1445. (10.1038/ng.2822) 24185512 PMC3903423

[bib260] Turner NC Ro J Andre F Loi S Verma S Iwata H Harbeck N Loibl S Huang Bartlett C Zhang K 2015 Palbociclib in hormone-receptor-positive advanced breast cancer. New England Journal of Medicine 373 209–219. (10.1056/NEJMoa1505270) 26030518

[bib261] Umekita Y Hiipakka RA Kokontis JM Liao S 1996 Human prostate tumor growth in athymic mice: inhibition by androgens and stimulation by finasteride. PNAS 93 11802–11807. (10.1073/pnas.93.21.11802) 8876218 PMC38139

[bib262] van de Velde CJ Rea D Seynaeve C Putter H Hasenburg A Vannetzel JM Paridaens R Markopoulos C Hozumi Y Hille ET 2011 Adjuvant tamoxifen and exemestane in early breast cancer (TEAM): a randomised phase 3 trial. Lancet 377 321–331. (10.1016/S0140-6736(10)62312-4) 21247627

[bib263] Vicier C Dieci MV Arnedos M Delaloge S Viens P Andre F 2014 Clinical development of mTOR inhibitors in breast cancer. Breast Cancer Research 16 203 (10.1186/bcr3618) 25189767 PMC3978635

[bib264] Waltering KK Urbanucci A Visakorpi T 2012 Androgen receptor (AR) aberrations in castration-resistant prostate cancer. Molecular and Cellular Endocrinology 360 38–43. (10.1016/j.mce.2011.12.019) 22245783

[bib265] Wang ZY Yin L 2015 Estrogen receptor alpha-36 (ER-alpha36): a new player in human breast cancer. Molecular and Cellular Endocrinology 418 193–206. (10.1016/j.mce.2015.04.017) 25917453

[bib266] Watson PA Chen YF Balbas MD Wongvipat J Socci ND Viale A Kim K Sawyers CL 2010 Constitutively active androgen receptor splice variants expressed in castration-resistant prostate cancer require full-length androgen receptor. PNAS 107 16759–16765. (10.1073/pnas.1012443107) 20823238 PMC2947883

[bib267] Welshons WV Jordan VC 1987 Adaptation of estrogen-dependent MCF-7 cells to low estrogen (phenol red-free) culture. European Journal of Cancer and Clinical Oncology 23 1935–1939. (10.1016/0277-5379(87)90062-9) 3436356

[bib268] Willson TM Henke BR Momtahen TM Charifson PS Batchelor KW Lubahn DB Moore LB Oliver BB Sauls HR Triantafillou JA 1994 3-[4-(1,2-Diphenylbut-1-enyl)phenyl]acrylic acid: a non-steroidal estrogen with functional selectivity for bone over uterus in rats. Journal of Medicinal Chemistry 37 1550–1552. (10.1021/jm00037a002) 8201587

[bib269] Wolf DM Jordan VC 1993 A laboratory model to explain the survival advantage observed in patients taking adjuvant tamoxifen therapy. Recent Results in Cancer Research 127 23–33. 8502820 10.1007/978-3-642-84745-5_4

[bib270] Wu YL Yang X Ren Z McDonnell DP Norris JD Willson TM Greene GL 2005 Structural basis for an unexpected mode of SERM-mediated ER antagonism. Molecular Cell 18 413–424. (10.1016/j.molcel.2005.04.014) 15893725

[bib271] Xiong R Patel HK Gutgesell LM Zhao J Delgado-Rivera L Pham TND Zhao H Carlson K Martin T Katzenellenbogen JA 2016 Selective Human Estrogen Receptor Partial Agonists (ShERPAs) for tamoxifen-resistant breast cancer. Journal of Medicinal Chemistry 59 219–237. (10.1021/acs.jmedchem.5b01276) 26681208 PMC4779956

[bib272] Yang X Guo Z Sun F Li W Alfano A Shimelis H Chen M Brodie AM Chen H Xiao Z 2011 Novel membrane-associated androgen receptor splice variant potentiates proliferative and survival responses in prostate cancer cells. Journal of Biological Chemistry 286 36152–36160. (10.1074/jbc.M111.265124) 21878636 PMC3195613

[bib273] Yao K Lee ES Bentrem DJ England G Schafer JI O’Regan RM Jordan VC 2000 Antitumor action of physiological estradiol on tamoxifen-stimulated breast tumors grown in athymic mice. Clinical Cancer Research 6 2028–2036. 10815929

[bib274] Yeh S Miyamoto H Shima H Chang C 1998 From estrogen to androgen receptor: a new pathway for sex hormones in prostate. PNAS 95 5527–5532. (10.1073/pnas.95.10.5527) 9576916 PMC20411

[bib275] Yu K Toral-Barza L Discafani C Zhang WG Skotnicki J Frost P Gibbons JJ 2001 mTOR, a novel target in breast cancer: the effect of CCI-779, an mTOR inhibitor, in preclinical models of breast cancer. Endocrine-Related Cancer 8 249–258. (10.1677/erc.0.0080249) 11566616

[bib276] Yue W Wang JP Conaway MR Li Y Santen RJ 2003 Adaptive hypersensitivity following long-term estrogen deprivation: involvement of multiple signal pathways. Journal of Steroid Biochemistry and Molecular Biology 86 265–274. (10.1016/S0960-0760(03)00366-2) 14623520

[bib277] Zhang L Altuwaijri S Deng F Chen L Lal P Bhanot UK Korets R Wenske S Lilja HG Chang C 2009 NF-kappaB regulates androgen receptor expression and prostate cancer growth. American Journal of Pathology 175 489–499. (10.2353/ajpath.2009.080727) 19628766 PMC2716950

[bib278] Zhao XY Malloy PJ Krishnan AV Swami S Navone NM Peehl DM Feldman D 2000 Glucocorticoids can promote androgen-independent growth of prostate cancer cells through a mutated androgen receptor. Nature Medicine 6 703–706. (10.1038/76287) 10835690

[bib279] Zhou Y Yau C Gray JW Chew K Dairkee SH Moore DH Eppenberger U Eppenberger-Castori S Benz CC 2007 Enhanced NF kappa B and AP-1 transcriptional activity associated with antiestrogen resistant breast cancer. BMC Cancer 7 59 (10.1186/1471-2407-7-59) 17407600 PMC1852565

